# Coordinated Role of Autophagy and ERAD in Maintaining Neuroendocrine Function by Preventing Prohormone Aggregation

**DOI:** 10.1002/advs.202411662

**Published:** 2025-03-07

**Authors:** Xuya Pan, Xing He, Su Wu, Na Xiong, Xinyu Hou, Heting Wang, Diane Somlo, Martin Spiess, Haiyang Wang, Jifeng Yang, Chunliang Li, Shasha Li, Wenbin Ma, Yanming Chen, Jun Cui, Ling Qi, Guojun Shi

**Affiliations:** ^1^ Department of Endocrinology and Metabolism Guangdong Provincial Key Laboratory of Diabetology & Guangzhou Municipal Key Laboratory of Mechanistic and Translational Obesity Research The Third Affiliated Hospital of Sun Yat‐sen University Guangzhou 510630 P. R. China; ^2^ MOE Key Laboratory of Gene Function and Regulation Guangdong Province Key Laboratory of Pharmaceutical Functional Genes State Key Laboratory of Biocontrol School of Life Sciences of Sun Yat‐sen University Guangzhou 510275 P. R. China; ^3^ Medical Research Center Sun Yat‐sen Memorial Hospital Sun Yat‐sen University Guangzhou 510120 P. R. China; ^4^ MOE Key Laboratory of Gene Function and Regulation State Key Laboratory of Biocontrol Guangzhou Key Laboratory of Healthy Aging Research School of Life Sciences Institute of Healthy Aging Research Sun Yat‐sen University Guangzhou 510275 P. R. China; ^5^ Department of Internal Medicine Massachusetts General Hospital Boston MA 02114 USA; ^6^ Biozentrum University of Basel Basel CH‐4056 Switzerland; ^7^ State Key Laboratory for Conservation and Utilization of Subtropical Agro‐Bioresources Guangdong Laboratory for Lingnan Modern Agriculture South China Agricultural University Guangzhou 510642 P. R. China; ^8^ Department of Tumor Cell Biology St. Jude Children's Research Hospital Memphis TN 38105 USA; ^9^ Department of Molecular Physiology and Biological Physics University of Virginia School of Medicine Charlottesville VA 22903 USA; ^10^ Sun Yat‐sen University Cancer Center State Key Laboratory of Oncology in South China Sun Yat‐sen University Guangzhou 510060 P. R. China

**Keywords:** autophagy, diabetes insipidus, endocrine neurons, ERAD, protein aggregates

## Abstract

Proteotoxicity induced by misfolded or aggregated proteins causes progressive neuronal damage. The endoplasmic reticulum (ER) protein quality control (ERQC) pathways are responsible for mitigating the accumulation of these misfolded or aggregated proteins, thus reducing proteotoxicity. Enhancing ERQC pathways is a promising strategy for treating neurodegenerative diseases. However, the mechanisms governing the initiation and degradation of misfolded or aggregated proteins in neurons remain largely unknown in vivo. In studying the maturation of proAVP in mouse AVP neurons, this study discovers that autophagy and ER‐associated degradation (ERAD) ERQC pathways collaborate to maintain proAVP maturation and protect AVP neuron survival against proteotoxicity. Autophagy deficiency in mouse AVP neurons leads to the late‐onset of diabetes insipidus. Mechanistically, autophagy selectively degrades mutant proAVP aggregates and endogenous HRD1 of the SEL1L‐HRD1 ERAD complex through FAM134B mediated ER‐phagy. HRD1 induction is responsible for reducing proAVP aggregation and maintaining AVP neuron function and survival under autophagy deficiency. Thus, autophagy and ERAD form a dual‐protection system that orchestrates prohormone maturation and endocrine neuron survival, providing new insights in the complexity of neuroendocrinology and the intrinsic mechanism of neurodegenerative diseases, with therapeutic potential in protein folding diseases.

## Graphical abstract

By studying the maturation mechanisms of vasopressin (AVP), this manuscript identifies FAM134B‐mediated ER‐phagy as a critical pathway for degrading both aggregated proAVP and the key ERAD component, HRD1. HRD1 induction mitigates proAVP aggregation and support AVP neuron function in the absence of autophagy. The findings establish autophagy and ERAD as a dual‐protection system for neuroendocrine proteostasis.

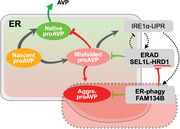

## 1 Introduction

Accumulation of protein aggregates is one of the leading factors in neurodegeneration, raiding the brain and killing neurons.^[^ ^1^ ^]^ Protein quality control (QC) systems are responsible for monitoring and triaging newly synthesized proteins to ensure proper folding, functioning, or timely degradation to prevent protein misfolding or aggregation.^[^ ^2^ ^]^ As large amounts of newly synthesized proteins are misfolding‐prone,^[^ ^3^ ^]^ the protein QC process must function as housekeeping mechanism to manage misfolded proteins. Protein QC involves the recognition, refolding, recycling, or clearance of misfolded proteins, which otherwise might accumulate or aggregate to induce loss of cellular function or cell death. However, how protein misfolding and aggregation are initiated and degraded in cells, and their link to disease progression in animal models, remains largely unclear.

A large number of peptide hormones regulate critical functions of physiological homeostasis in mammals, which are first synthesized in the endoplasmic reticulum (ER) as precursor proteins (prohormones) and require stringent surveillance by the ER protein quality control (ERQC) machineries. Thus, protein homeostasis in the ER is crucial for normal cellular function and organismal health and is regulated by three major protein quality control mechanisms: unfolded protein response (UPR), ER‐associated degradation (ERAD), and autophagy.^[^ ^4^, ^5^, ^6^ ^]^ ERAD is the primary pathway for clearing misfolded or unfolded proteins in the ER via proteasomal degradation,^[^ ^6^, ^7^ ^]^ with critical physiological functions.^[^ ^8^, ^9^, ^10^, ^11^, ^12^ ^]^ In mammals, HRD1 is the primary ER‐resident E3 ligase in complex with SEL1L to degrade misfolded ER proteins.^[^ ^6^, ^12^, ^13^, ^14^ ^]^ Autophagy is an intracellular degradation system responsible for maintaining organelle function by degrading excess or defective proteins or portions of organelles.^[^ ^15^ ^]^ ER‐phagy, a subtype of autophagy, is responsible for the clearance of ER‐accumulated protein aggregates.^[^ ^16^, ^17^, ^18^, ^19^ ^]^ Most previous studies investigated the roles of ERAD or autophagy in vitro individually, and suggested that these systems acted in parallel to reduce misfolded or aggregated proteins to maintain ER proteostasis.^[^ ^2^, ^20^ ^]^ Recent in vivo studies on the roles of ERAD and autophagy demonstrated distinct physiological functions of the two systems in a tissue‐dependent manner.^[^ ^8^, ^9^, ^21^ ^]^ Deficiency in ERQC pathways can lead to various diseases, including diabetes mellitus, neurodegenerative diseases, obesity, and cystic fibrosis.^[^ ^6^ ^]^ However, how the capacities of or cross‐talk between ERQC pathways are involved in preventing or clearing protein aggregation, as well as in disease progression under environmental, genetic, behavioral, or aging‐related insults, remains to be investigated.^[^ ^22^, ^23^ ^]^

Prohormones exit the ER upon reaching their native folding state, and undergo additional proteolytic processing. In the trans‐Golgi, some of the prohormones or their products self‐aggregate to form secretory granules for regulated secretion, which likely makes them aggregation‐prone in the ER.^[^ ^22^ ^]^ Previous studies have suggested that arginine vasopressin (AVP) maturation represents a unique model for studying the ERQC of prohormones and their physiological functions. AVP is a nonapeptide hormone derived from a 145 amino acid prohormone (proAVP) with eight disulfide bonds in AVP‐producing neurons of the hypothalamus.^[^ ^24^, ^25^, ^26^, ^27^, ^28^ ^]^ ProAVP is synthesized and folded into its native conformation in the ER and packed into secretory granules during its transition to the Golgi apparatus. AVP, Neurophysin II, and Copeptin are then cleaved from proAVP in the vesicles during axonal transport to the pituitary gland, which further controls the systemic water balance by stimulating water resorption in the kidney. Dehydration‐induced osmotic stress induces the release of AVP granules into the blood from axon endings in the pituitary.^[^ ^24^ ^]^ More than 50 missense mutations in proAVP have been identified in humans, causing familial neurohypophyseal diabetes insipidus (FNDI), a dominant disease in which AVP production fails, resulting in excess urination and free water loss.^[^ ^29^ ^]^ Many of these mutations are associated with proAVP trapped in the ER as disulfide‐linked fibrillar aggregates.^[^ ^24^, ^30^ ^]^ However, it has been shown that even wild‐type (WT) proAVP is misfolding‐prone in the ER both in vitro and in vivo,^[^ ^24^ ^]^ and how proAVP aggregates are formed and cleared, as well as its detrimental effect on neuronal function and degeneration, remains to be explored. Thus, proAVP maturation provides a unique and ideal model to study protein QC mechanisms in protein misfolding and aggregation related diseases in vivo by visualizing the maturation process of proAVP in AVP neurons.

Both mutant and WT misfolded proAVP proteins undergo proteasomal degradation.^[^ ^24^, ^31^ ^]^ The SEL1L‐HRD1 ERAD pathway degrades misfolded WT proAVP to prevent the formation of aberrant disulfide bonds between proAVP molecules.^[^ ^24^ ^]^ Loss of *Sel1L* in AVP neurons causes ER retention and aggregation of proAVP and the development of a diabetes insipidus phenotype in mice. However, loss of water homeostasis and accumulation of proAVP aggregates in AVP neurons with *Sel1L* deficiency were gradual rather than immediate,^[^ ^24^ ^]^ suggesting that other mechanisms might be involved in adapting to ERAD deficiency or clearing misfolded or aggregated proAVP. Earlier studies have shown that in mice with the FNDI mutant proAVP, autophagy was activated, and autophagy‐associated neuronal death was induced in AVP neurons when subjected to osmotic stress.^[^ ^26^, ^32^, ^33^ ^]^ However, the ERQC mechanisms on how WT proAVP aggregates form and accumulate under physiological conditions, as well as the pathological roles of the proAVP aggregates, remain largely uncharacterized in vivo.

In this study, the signaling pathways involved in the dehydration response in AVP neurons were analyzed by transcriptomics, and the interactomes of WT and FNDI mutant proAVP in vitro were demonstrated using TurboID.^[^ ^34^, ^35^ ^]^ By generating mice with AVP neuron‐specific deficiency of autophagy and ERAD, we analyzed the physiological roles of ERQC pathways in maintaining endogenous AVP maturation and reported the cooperative role of autophagy and ERAD in orchestrating the maturation of proAVP under physiological or stress conditions. Along with proAVP maturation analysis under compound deficiency of autophagy and ERAD both in vivo and in vitro, we propose that autophagy and ERAD form a dual‐protective safeguard system in AVP neurons to maintain AVP production, neuronal survival, and water homeostasis.

## 2 Results

### 2.1 Bioinformatic Analysis Reveals the Involvement of Autophagy and the ER Stress Response in Neuroendocrine Neurons During Development

**Figure 1:**
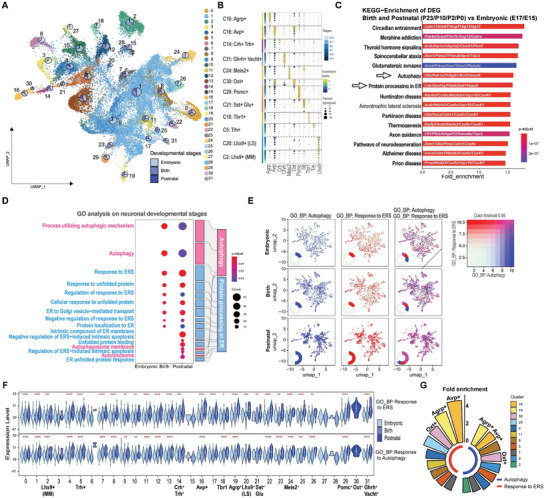
Autophagy and ER stress response pathways are activated during the development of endocrine neurons from the embryonic to postnatal stages. A) UMAP visualization of single‐cell RNA‐seq data from mouse hypothalamic neurons (GSE132730). A total of 34580 neuronal cells were sorted and grouped into 32 (C0‐C31) clusters. Hypothalamic samples from E15.5 (embryonic day, *n* = 8), E17.5 (*n* = 7), P0 (postnatal day, *n* = 4), P2 (*n* = 4), P10 (*n* = 3), and P23 (*n* = 3) were used. The samples were then grouped into embryonic (E15.5 and E17.5), birth (P0 and P2), and postnatal (P10 and P23) stages, with cell number percentages shown as pie graphs. B) Bubble diagram showing gene expression patterns of the selected 12 hypothalamic neuron clusters, including 11 endocrine neurons and 2 non‐endocrine neurons with recognized markers, with 6 developmental stages, 11 marker gene expression levels, and the ratio of cells with detected marker genes were shown. C) Functional annotation and pathway analysis of the differentially expressed genes (DEGs) using KEGG enrichment analysis, by comparing neurons in the embryonic and postnatal stages with the top DEGs shown. D) Bubble Sankey Diagram showing the detailed Gene Ontology (GO) analysis of DEGs of neurons by statistically comparing postnatal stages (P0‐P23) with embryonic stages (E15.5‐E17.5). The biological process (BP) pathways from (A) were demonstrated in detailed pathways. E) AddModuleScore analysis of all neurons in 3 developmental stages showing the “autophagy” and “Response to ERS” pathways as demonstrated in (D), as well as their co‐expression pattern. F) Violin plots showing the scores of the 32 clustered neurons with activation of “Autophagy” and “Response to ERS” pathways. Statistical analysis was performed by comparing postnatal stages (P0‐P23) with embryonic stages (E15.5‐E17.5) using *Student's test*. *p < 0.05; **p < 0.01; ***p< 0.001; ****p < 0.0001. G) GO‐BP enrichment showing the ranking of “*Autophagy*” and “*Response to ERS*” pathways across 32 neuronal clusters during development.

Single‐cell RNA‐seq analysis provides unique information on neuronal development and function.^[^ ^36^ ^]^ To understand the role of protein quality control pathways in maintaining neuronal proteostasis and development, public scRNA‐seq data from mouse hypothalamus before and postnatally were re‐analyzed.^[^ ^36^ ^]^ Neuronal cells were extracted from the hypothalamic cell pool accordingly,^[^ ^36^ ^]^ followed by clustering analysis and Uniform Manifold Approximation and Projection (UMAP) visualization (**Figure** **1**A). 32 clusters of neuronal cells were identified, while 10 clusters of neuroendocrine neurons and 2 clusters of non‐endocrine neurons were shown to demonstrate and confirm the gene expression patterns after clustering (Figure 1B). Kyoto Encyclopedia of Genes and Genomes (KEGG) analysis of differentially expressed genes (DEGs) between neurons before and after birth identified “*autophagy*” and “*protein processing in ER*” as potentially important for neuronal development, which are critical for the ERQC of peptide hormones (Figure 1C). The two pathways were further analyzed by Bubble Sankey Diagram showing the Gene Ontology (GO) analysis of DEGs of neurons by statistically compared postnatal stages (P0‐P23) with embryonic stages (E15.5‐E17.5) (Figure 1D). It showed that “*Autophagy*” and “*Response to ER stress (ERS)*” pathways were activated during neuronal development (Figure 1D), indicating that the increased burden of protein misfolding during neuronal development might trigger the activation of “*Autophagy*” and “*Protein processing in ER*” in a synergistic manner (Figure 1E). The potential involvement of the two ERQC pathways in neuronal development was confirmed in the 32 clusters of neurons by GO analysis between postnatal (P10 and P23) and embryonic stages (E15 and E17) (Figure 1F). Fold‐change enrichment analysis showed that AVP neurons, which regulate water homeostasis and blood pressure, were ranked as the most affected cluster of the 32 neuron clusters that could be affected by “*autophagy*” and “*protein processing in ER*” pathways during neuronal development (Figure 1G). We conclude that autophagy and ER stress response pathways, which are key ERQC pathways, may play important roles in hypothalamic neuron development.

### 2.2 Autophagy is Involved in ProAVP Maturation Both In Vivo and In Vitro

**Figure 2:**
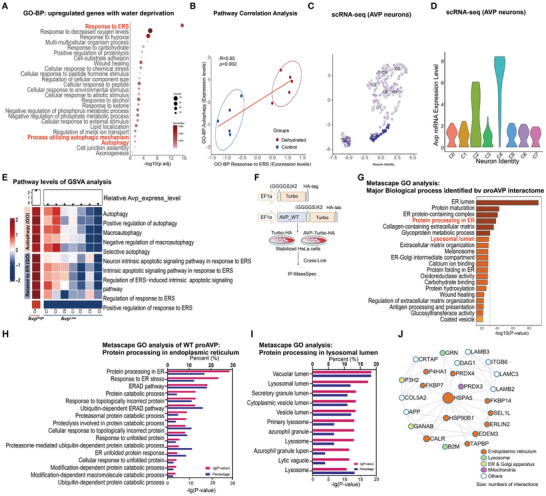
Autophagy and ERAD pathways are involved in proAVP maturation both in vivo and in vitro. A) Bubble plot showing the GO‐BP analysis of upregulated genes in isolated hypothalamus from rats under 72 h water deprivation (GSE175461), with the top 25 pathways listed. B) Correlation analysis of the pathway mean hypothalamic expression values of “Autophagy” and “Response to ERS” pathways between dehydrated and control groups. C,D) UMAP visualization of *Avp* mRNA expressing cells of the postnatal stages from the single cell RNA‐seq data, which were grouped into 8 clusters (C), and *Avp* mRNA expression levels of each cluster were shown as violin plots (D). E) GSVA analysis was performed on a selection of 30 cells from each identified postnatal hypothalamic‐neuron cluster, targeting autophagy and ERS‐related pathways from the GO database. F) Diagram of the sequence elements in the vectors for proAVP‐Turbo fusion protein expression, and workflow of the TurboID assay for identifying proAVP proximal proteins. G–I) GO analysis of proAVP (WT) enriched preys (G), with preys functioning in protein processing in ER (H) and in protein processing in lysosomal lumen (I) were shown, respectively. J) Enriched proteins network from proAVP (WT) that have been identified by TurboID. Colors represent subcellular localization and sizes of the dots represents the fold change. Statistical analysis were performed with 3 samples of each group (G–J).

The regulatory mechanism on the activation of ERQC pathways in neurons remains largely unclear. The synthesis, maturation and degradation of proAVP in AVP neurons provide an ideal model to study the ERQC mechanisms of hormone production and neuroendocrine function. Thus, we analyzed the public transcriptomic data from the hypothalamic supraoptic nucleus (SON) of rats after dehydration for 72 h, which mainly activates the synthesis and secretion of AVP.^[^ ^37^ ^]^ Interestingly, both “response to ERS” and “autophagy” pathways which are responsible for maintaining ER protein homeostasis, were activated by dehydration (**Figure** **2**A). Further analysis also revealed that autophagy and ERAD pathways were synergistically activated under dehydration (Figure 2B), which might due to the increased proAVP synthesis under dehydration. To explore the physiological functions of autophagy and ERS pathways in AVP neurons, single‐cell RNA sequencing data were re‐analyzed again for *Avp* mRNA expression.^[^ ^36^ ^]^ Postnatal neurons expressing *Avp* were clustered into 8 groups using UMAP visualization (Figure 2C,D). Gene set variation analysis (GSVA) was then conducted on 30 cells from each cluster to assess autophagy and ERS‐related pathways from the GO database. This analysis revealed that postnatal neurons formed seven distinct clusters. Notably, neurons in the postnatal cluster 2 (*Avp^High^*) displayed significantly higher activity in autophagy and ERS pathways, as shown in the heatmap (Figure 2E). These data suggest that neurons with increased synthesis of proAVP require the activation of autophagy and ERS pathways to maintain ER homeostasis and AVP production.

To understand the detailed mechanisms of ERQC pathways in maintaining proAVP maturation, TurboID was performed to identify proAVP proximal proteins (Figure 2F), which is a proximity‐based labeling technique for detecting protein‐protein interaction.^[^ ^35^ ^]^ As expected, proteins in the ER were among the top enriched proteins of the WT proAVP interactome (Figure 2G,H). Interestingly, proteins in the lysosomal lumen were also significantly enriched, suggesting the involvement of ER‐to‐lysosome‐associated degradation (ERLAD) or ER‐phagy to segregate certain forms of proAVP for clearance (Figure 2G,I).^[^ ^19^ ^]^ The enriched proteins identified also included the key ERAD proteins (like SEL1L) and lysosomal proteins (like GRN) (Figure 2J), which confirms the previous findings.^[^ ^24^, ^26^, ^38^ ^]^ These results indicate that proAVP in AVP neurons is misfolding prone, and both ERAD and autophagy pathways are involved in the ERQC of proAVP maturation.^[^ ^25^ ^]^

### 2.3 Autophagy Deficiency in AVP Neurons Leads to Late‐Onset Diabetes Insipidus

**Figure 3:**
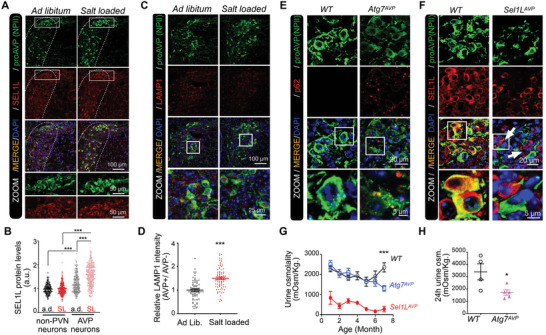
Autophagy deficiency in AVP neurons leads to late‐onset of diabetes insipidus. A–D) Representative fluorescent images of proAVP and SEL1L staining (A), or LAMP1 staining (C), in the PVN of *WT* mice at 8–12 weeks of age, fed on regular water or 2% salty water for 7 days. Quantification of SEL1L intensity (B), or LAMP1 intensity (D) in AVP neurons and non‐PVN neurons were shown. E,F) Representative immunostaining images for p62 and proAVP in PVNs of *Atg7^AVP^* mice (E), and for SEL1L and proAVP of *Sel1L^AVP^* mice (F), with their littermate controls. G‐H) The osmolality levels of freshly collected urine samples at indicated ages of *Atg7^AVP^* and *WT* mice (G), and urine samples collected for 24 h (H) in mice around 6–8 months of age. Values, mean ± SEM. ^***^ *p* < 0.001 by *one‐way ANOVA* (B); ^*^ *p* < 0.05; ^***^ *p* < 0.001 by *Student's t‐test* (D, G, H). Data represents at least 3 mice in each group, or each mouse was represented by dots (H).

To study the mechanistic role of autophagy in prohormone maturation in endocrine neurons in vivo, WT mice were fed with salty water for indicated days (Figure S1A,B, Supporting Information). Protein levels of SEL1L and LAMP1 in the paraventricular nucleus (PVN) of hypothalamus were examined, which indicates ERAD and autophagy activities, respectively.^[^ ^24^, ^39^ ^]^ It showed that SEL1L protein levels in AVP neurons were significantly increased compared with adjunct non‐PVN neurons, and increased in AVP neurons after salty water feeding induced dehydration (**Figure** **3**A,B). LAMP1 protein levels in AVP neurons were increased compared to non‐AVP neurons in the PVN under dehydration (Figure 3C,D). These data confirm the previous analysis (Figures 1 and 2) that ERAD and autophagy are activated under osmotic stress in AVP neurons.

To explore the role of autophagy in proAVP maturation, we generated mice with AVP‐neuron‐specific deficiency of *Atg7* (*Atg7^AVP^*), a factor required for autophagosome formation,^[^ ^40^ ^]^ and analyzed side by side with *Sel1L^AVP^* mice generated as previously described.^[^ ^24^ ^]^ Deletion of *Atg7* was confirmed by the accumulation of the autophagy substrate p62 in AVP neurons (Figure 3E),^[^ ^41^ ^]^ and deletion of *Sel1L* was confirmed by SEL1L staining (Figure 3F). Both *Atg7^AVP^* and *Sel1L^AVP^* mice showed comparable growth curves with WT mice (Figure S1C, Supporting Information). While *Sel1L^AVP^* mice developed diabetes insipidus in 3 weeks postnatally, including polyuria and polydipsia, *Atg7^AVP^* mice showed unchanged water intake, urine output, and urine osmolality compared with WT mice around 8 weeks postnatally (Figure S1D–G, Supporting Information). Interestingly, *Atg7^AVP^* mice showed reduced urine osmolality around 6 months of age (Figure 3G,H). These data indicate that mice with AVP neuron‐specific autophagy deficiency develop late‐onset diabetes insipidus,^[^ ^42^ ^]^ and suggest a potential mechanism for how AVP neurons adapt to autophagy deficiency for maintaining AVP production.

### 2.4 Autophagy Deficiency in AVP Neurons Leads to Reduced ProAVP Protein Levels and Elevated ER Chaperone Levels

**Figure 4:**
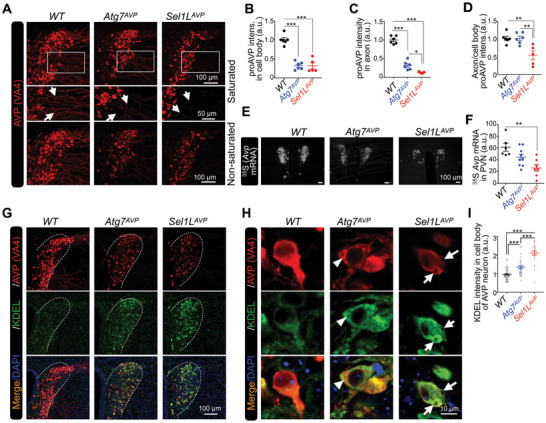
Autophagy deficiency in AVP neurons results in elevated ER stress. A) Representative immunostaining images of proAVP in the PVN of *Atg7^AVP^*, *Sel1L^AVP^*, and *WT* control mice at 3–5 weeks of age. Both saturated and non‐saturated images of proAVP staining were shown for visualizing axonal proAVP. B–D) Quantification of proAVP intensity in cell bodies (B) and axons (C), as well as the ratio of proAVP in axons to cell bodies (D), was performed in the PVN of *Atg7^AVP^*, *Sel1L^AVP^*, and *WT* control mice at 3–5 weeks of age. E,F) Representative images of ^35^S labeled RNA probe based in situ hybridization against proAVP mRNA in the PVNs of *Atg7^AVP^*, *Sel1L^AVP^*, and *WT* control mice at 9–15 weeks of age (E), with radiometric quantifications shown in (F). Each dot represents the staining intensity of either left or right region of the PVN as indicated. G–I) Representative immunostaining images (G) and higher resolution images (H) of proAVP and KDEL staining in the PVNs of *Atg7^AVP^*, *Sel1L^AVP^*, and *WT* control mice, with quantification of KDEL protein intensity shown in (I). Each dot represents the staining intensity of one field. Arrowheads and arrows indicate the interesting staining patterns of proAVP in *Atg7^AVP^* or *Sel1L^AVP^*, respectively. Values, mean ± SEM. ^*^ *p* < 0.05; ^**^ *p* < 0.01; ^***^ *p* < 0.001 by *one‐way ANOVA*. Data represents at least 3 mice in each group, or each mouse was represented by one dot (B‐D).

To understand how AVP neurons adapt to autophagy deficiency before developing diabetes insipidus, the distribution patterns of proAVP in the cell bodies and axons of AVP neurons were examined by immunostaining. ProAVP signals in neuronal cell bodies, which represent immature proAVP, were reduced both in *Atg7^AVP^* and *Sel1L^AVP^* mice around 1 month of age (**Figure** **4**A,B). Interestingly, axon localized proAVP, which was recognized as folded and matured proAVP, was reduced in *Atg7^AVP^* mice compared with WT mice, while almost absent in *Sel1L^AVP^* mice (Figure 4A,C). Quantitatively, the distribution ratios of proAVP between axons and cell bodies were comparable in *Atg7^AVP^* and WT mice, but significantly reduced in *Sel1L^AVP^* mice (Figure 4D). The *Avp* mRNA level, as measured by in situ hybridization with ^35^S‐labeled probes (Figure 4E), was reduced in *Sel1L^AVP^* mice but not reduced yet in *Atg7^AVP^* mice compared to *WT* (Figure 4F). To analyze the ER homeostasis which is required for efficient proAVP synthesis and maturation, PVN sections were stained with proAVP, together with KDEL‐domain proteins which represent ER chaperones like BiP and GRP94. KDEL signal intensity in AVP neurons of *Atg7^AVP^* mice was increased compared to WT mice, but not as much increased as in *Sel1L^AVP^* mice (Figure 4G–I). Interestingly, AVP neurons of *Atg7^AVP^* mice demonstrated a pattern of “island” like proAVP signal surrounded by KDEL, but not in *Sel1L^AVP^* mice (Figure 4H), suggesting the different content or folding status of proteins surrounded by ER chaperones. These data indicate that deficiency of autophagy leads to increased ER stress response but comparable maturation efficiency of proAVP in AVP neurons. Our results suggest that even with functional ERAD, autophagy is still very important for maintaining ER homeostasis and neuroendocrine function under physiological conditions.

### 2.5 Autophagy Deficiency in AVP Neurons Leads to Increased Susceptibility to Osmotic Stress Induced Diabetes Insipidus

**Figure 5:**
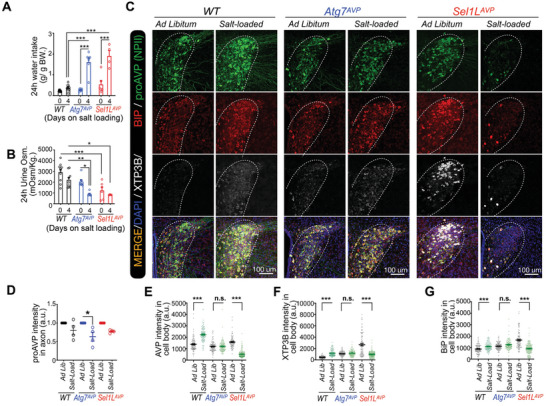
Osmotic stress accelerates the progression of diabetes insipidus in mice with *Atg7* deficiency in AVP neurons. A,B) 24 h water intake (A) and urine volume (B) of *Atg7^AVP^*, *Sel1L^AVP^*, and wildtype control mice fed on regular water or 2% salty water for 4 days. Male and female mice at around 12–14 weeks of age were used. 4–7 mice in each group. C–G) Representative fluorescent images of proAVP, BiP, and XTP3B staining in PVN of *Atg7^AVP^*, *Sel1L^AVP^*, and wild‐type control mice fed with regular water or 2% salty water for 7 days (C). Quantification of AVP intensity in axonal regions (D), and intensities in individual cell bodies by staining with AVP (E), XTP3B (F), and BiP (G) in mice with indicated genotypes were shown as indicated. Data represents at least 3 mice in each group. Each dot represents the staining intensity of one field (E‐G). Mice number was indicated by dots in (A, B), and *n* = 3–5 mice in each group in (C‐G). Values, mean ± SEM. ^*^ *p* < 0.05, ^**^ *p* < 0.01; ^***^ *p* < 0.001 by two‐way ANOVA.

To explore how AVP neurons adapt to autophagy deficiency under disease settings, proteostasis of AVP neurons was analyzed under osmotic stress by feeding with salty water, which induces the accumulation of misfolded proAVP,^[^ ^24^ ^]^ and activation of autophagy and ERAD pathways (Figures 2 and 3). Thus, this model would provide the clues for understanding the pathophysiological roles of autophagy or ERAD deficiency in AVP neurons. Compared with WT littermates, *Atg7^AVP^* mice (aged around 3 months) showed increased water intake (**Figure** **5**A) and reduced urine osmolality (Figure 5B) when fed with salty water, to a similar extent to *Sel1L^AVP^* mice. Immunostaining revealed that proAVP protein levels in axons of AVP neurons were reduced in *Atg7^AVP^* mice under salty water feeding compared with the control condition (Figure 5C,D). However, proAVP protein levels in axons of AVP neurons were comparable between the two conditions in either WT or *Sel1L^AVP^* mice, even though the basal expression levels were different among the 3 genotypes of mice. These results indicate that autophagy in AVP neurons is required for adaption to osmotic stress in mice.

To further analyze the proteostasis in AVP neurons under osmotic stress, ER chaperone protein levels were analyzed. ProAVP protein levels in the cell bodies of AVP neurons in WT mice were increased when fed with salty water (Figure 5E), as well as increased protein levels of BiP and XTP3B, which are ER chaperones (Figure 5F,G), indicating the elevated ER stress response to increased proAVP synthesis. However, there was no obvious increase in proAVP and XTP3B in the cell bodies of AVP neurons in *Atg7^AVP^* mice after salty water feeding (Figure 5E,F), although increased BiP was observed (Figure 5G). It is noteworthy to mention that proAVP, BiP, and XTP3B intensities in AVP neurons of *Sel1L^AVP^* mice were even decreased under salty water feeding compared with ad libitum (Figure 5E–G), suggesting the exhausted response in the ER under osmotic stress with ERAD deficiency. These data indicate that the response to osmotic stress was impaired with either autophagy or ERAD deficiency in AVP neurons.

### 2.6 FAM134B‐Dependent ER‐Phagy Mediates the Degradation of proAVP Aggregates

**Figure 6:**
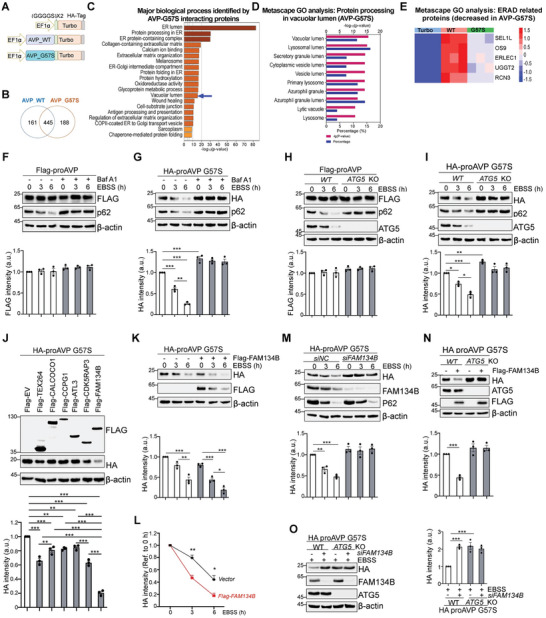
Autophagy is responsible for degrading misfolded proAVP aggregates through FAM134B dependent ER‐phagy. A) Diagram of the sequence elements in the vectors for G57S mutant proAVP‐Turbo fusion protein expression in addition to previously shown in Figure 2F. B) Venn diagram showing the numbers of unique and overlapping interacting proteins captured by WT and G57S proAVP. 3 samples in each group were performed and analyzed. C,D) GO analysis of G57S mutant proAVP enriched interacting proteins (C), with the “Vacuolar lumen” pathway‐related proteins shown (D). E) Heatmap of downregulated ERAD‐related proteins in G57S mutant proAVP compared with WT proAVP. Statistical analysis were performed with 3 samples of each group (B‐E). F,G) Western blot analysis of WT (F) or G57S mutant (G) proAVP overexpressed in HeLa cells under normal or starvation conditions (cultured in EBSS), and treated with Bafilomycin A1 (Baf A1, autophagy inhibitor, 0.2 µm) at indicated time. H,I) Western blot analysis of WT (H) or G57S mutant (I) proAVP stability cultured with EBSS at indicated time in HEK293T cells. J) Western blot analysis of G57S mutant proAVP protein levels cultured with EBSS in HeLa cells overexpressed with indicated ER‐phagy receptors. K–M) Western blot analysis of G57S mutant proAVP stability cultured with EBSS for the indicated time in HeLa cells transfected with *Flag‐FAM134B* expression construct (K, with protein stability quantified in L) or *siFAM134B* (M). N–O) Western blot analysis G57S mutant proAVP stability cultured with EBSS in WT or *ATG5* deficient HEK239T cells, with *Flag‐FAM134B* expression construct (N, with quantification shown below) or *siFAM134B* (O, with quantification shown on the right). Values, mean ± SEM. ^*^ *p* < 0.05; ^**^ *p* < 0.01; ^***^ *p* < 0.001 by *one‐way* ANOVA. Data represent at least 3 independent experiments (F‐O).

To understand why autophagy deficiency impairs the response to osmotic stress in AVP neurons, biochemical analysis on the clearance of proAVP at different folding statuses was performed in vitro. Autophagy pathway is involved in clearing protein aggregates in the ER, which was named ER‐phagy.^[^ ^19^, ^25^ ^]^ ProAVP is misfolded and aggregation‐prone, with hydrophobic domains in two ends that could form amyloid‐like fibrillary aggregates when misfolded (Figure S2A,B, Supporting Information).^[^ ^30^ ^]^ To explore the mechanistic roles of ERQC pathways in degrading native, misfolded or aggregated forms of proAVP, TurboID was performed with aggregation‐prone G57S mutant proAVP, which represents the accumulation of misfolded and aggregated proAVP (**Figure** **6**A). Together with the previous TurboID analysis with WT proAVP (Figure 2F–J), it showed that 445 proteins were identified in the interactome of WT and G57S mutant proAVP in common, while 161 and 188 proteins were uniquely identified in the proximity of WT or G57S mutant proAVP, respectively (Figure 6B). Bioinformatics analysis indicated that the proximal proteins with G57S mutant proAVP were enriched with ER and vacuolar lumen‐related proteins (Figure 6C,D; Figure S2C, Supporting Information). The differentially enriched proteins between WT and G57S mutant proAVP and their interacting network were shown (Figure S2D,E, Supporting Information), which were related to synapse organization and amyotrophic lateral sclerosis (Figure S2F,G, Supporting Information). Unexpectedly, ERAD core components, including SEL1L and OS9,^[^ ^43^, ^44^ ^]^ were significantly reduced in the interactome of G57S mutant compared with WT proAVP (Figure 6E). These data indicate that misfolded proAVP aggregates might be resistant to ERAD degradation.

To study whether autophagy was involved in the degradation of native or misfolded proAVP, which is one of the two major protein clearance pathways in the ER, the stability of WT and G57S proAVP proteins under normal and starvation conditions were analyzed in vitro. Autophagy activation by starvation (incubation in nutrient‐free EBSS buffer) did not reduce the protein stability of WT proAVP, while p62 protein levels were reduced and reversed by the autophagy inhibitor Baf A1 (Figure 6F, with quantification shown below). These data demonstrated the successful induction and inhibition of autophagy. Interestingly, the G57S mutant proAVP was degraded upon EBSS treatment in a process blocked by Baf A1 (Figure 6G). To confirm this phenomenon, *ATG5* deficiency in HEK293T cells, which is another essential gene for autophagosome formation, inhibited the starvation‐induced degradation of G57S mutant proAVP, while WT proAVP remained intact (Figure 6H,I), confirming the involvement of autophagy in degrading misfolded proAVP aggregates.

To investigate the mechanism of how proAVP aggregates in the ER are cleared by autophagy, 6 of the well‐recognized ER‐phagy receptors, including TEX264, CALCOCO1, CCPG1, ATL3, CDK5RAP3, and FAM134B,^[^ ^15^ ^]^ were individually overexpressed in HeLa cells together with G57S mutant proAVP. Our data showed that the G57S mutant proAVP was the most obviously reduced with FAM134B overexpression (Figure 6J). Thus, we further showed that overexpression of FAM134B promoted the degradation of the G57S mutant proAVP (Figure 6K,L), and siRNA silencing of FAM134B (Figure S2H, Supporting Information) attenuated G57S mutant proAVP degradation (Figure 6M). The role of FAM134B in promoting the ER‐phagy‐dependent degradation of G57S mutant proAVP was blocked in *ATG5*‐KO cells by either gain‐of‐function (Figure 6N) or loss‐of‐function of FAM134B (Figure 6O). Taken together, these data indicate that misfolded proAVP aggregates, but not the natively folded proAVP, could be degraded through the FAM134B mediated ER‐phagy.

### 2.7 Autophagy Activation Degrades HRD1 Through the FAM134B‐Dependent ER‐Phagy Pathway

**Figure 7:**
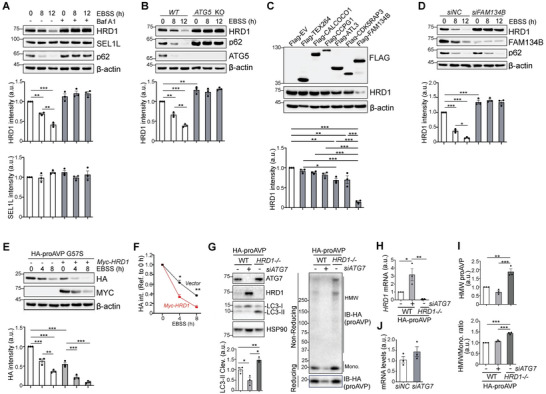
Autophagy degrades HRD1 through the FAM134B‐dependent ER‐phagy pathway. A,B) Western blot analysis of HRD1 stability under normal or starvation (EBSS media) conditions, with or without Baf A1 treatment in HeLa cells (A), or *ATG5* knocked‐out HEK293T cells (B) at indicated time. C) Western blot analysis of HRD1 protein levels cultured with EBSS in HeLa cells overexpressed with indicated ER‐phagy receptors. D) Western blot analysis of HRD1 stability cultured with EBSS at indicated time in HeLa cells with siRNA‐mediated knocking‐down of FAM134B. E,F) Western blot analysis of the G57S mutant proAVP stability cultured with EBSS at indicated time in HeLa cells overexpressed with vector or Myc‐tagged HRD1, with proAVP protein stability quantified in (E‐F). G–J) Western blot analysis showing the protein levels of ATG7, HRD1, LC3I/II, and proAVP in *ATG7* deficient (by siRNA) or *HRD1* deficient (by CRISPR‐Cas9) HEK293T cells (G). Quantification of HRD1, high molecular weight (HMW) proAVP, and ratio of HWM with Monomeric proAVP were shown (H‐I). HRD1 mRNA levels with ATG7 deletion was shown (J). Values, mean ± SEM. ^*^ *p* < 0.05; ^**^ *p* < 0.01; ^***^ *p* < 0.001 by *one‐way ANOVA*. Data represent at least 3 independent experiments (F‐O).

To investigate whether ERAD adapts to autophagy deficiency in maintaining ER homeostasis, core ERAD protein levels were examined.^[^ ^24^ ^]^ Surprisingly, it showed that HRD1, but not SEL1L, was degraded with activated autophagy at indicated time points (**Figure** **7**A). Consistently, either inhibition of autophagy by Bafilomycin A1 (Baf A1) (Figure 7A) or by ATG5 deletion (Figure 7B) attenuated HRD1 degradation. To investigate the mechanism of how HRD1 in the ER is degraded by autophagy, 6 of the well‐recognized ER‐phagy receptors were overexpressed in vitro. Interestingly, FAM134B overexpression showed the most obvious reduction in endogenous HRD1 protein level under starvation (Figure 7C). Further, knocking‐down of FAM134B abolished HRD1 reduction under starvation condition (Figure 7D). To test whether elevated HRD1 protein level would promote the clearance of misfolded proteins, G57S mutant proAVP construct was co‐transfected with HRD1 cDNA or control, showing the reduced protein levels of G57S mutant proAVP by elevated HRD1 under either basal or EBSS induced autophagic activation conditions (Figure 7E,F). The elevated protein level of HRD1 was also confirmed in ATG7 deficient HEK293T cells with intact mRNA expression level (Figure 7G,H). LC3‐II level, which is the cleaved form of LC3‐I showing autophagic activation, was reduced with ATG7 deficiency while increased with HRD1 deficiency, indicating the activation of autophagy with HRD1 deficiency. Indeed, HMW form of WT proAVP indicated that HRD1 deficiency induced the accumulation of misfolded proAVP and compromised folding efficiency, while ATG7 deficiency showed reduced trend of proAVP misfolding but intact folding efficiency (Figure 7I). Activation of autophagy with ERAD deficiency was also confirmed in AVP neurons. Increased LAMP1 and proAVP co‐localization efficiency was observed in *Sel1L^AVP^* mice compared with WT controls (Figure S3A,B, Supporting Information). Interestingly, the colocalization efficiency is negatively associated with ER stress levels of individual AVP neurons indicated by XTP3B protein chaperone levels (Figure S3C, Supporting Information), suggesting that autophagy activation could reduce ER stress in AVP neurons by degrading misfolded protein aggregates. These data indicate that autophagy deficiency attenuates FAM134B mediated degradation of HRD1 by ER‐phagy, thus adaptively increasing ERAD activity to degrade misfolded proAVP and improving proAVP maturation efficiency. These results show that the degradation of G57S mutant proAVP was accelerated in the presence of endogenously elevated or ectopically expressed HRD1, indicating the dual‐protective ERQC mechanism comprised of ER‐phagy and ERAD that elevated ERAD activity with autophagy deficiency could promote the degradation of misfolded proAVP.

### 2.8 The Protective Role of ERAD Activation Under Autophagy Deficiency Declined with Aging in Maintaining ProAVP Maturation

**Figure 8:**
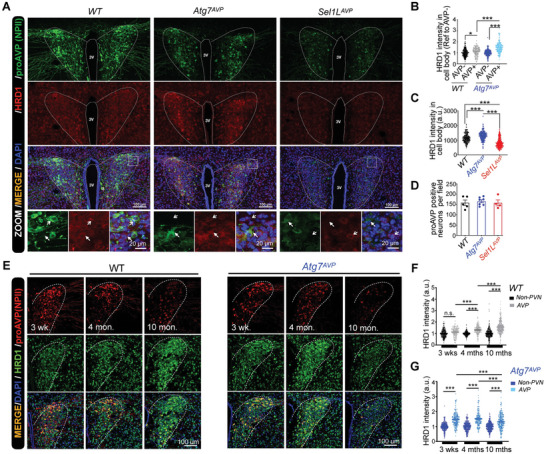
*Atg7^AVP^* mice fails to maintain water homeostasis with aging, which is associated with impaired HRD1 induction in AVP neurons. A–D) Representative fluorescent images of proAVP and HRD1 in the PVN of *Atg7^AVP^*, *Sel1L^AVP^*, and wildtype control mice at around 4 weeks of age, with white arrows indicating AVP neurons with differential HRD1 expression levels (A). Quantification of HRD1 intensities in AVP positive and negative neurons in the PVN region of *WT* and *Atg7^AVP^* mice was shown (B). HRD1 intensity in the cell body of AVP neurons in the PVN region (C), as well as the numbers of AVP positive neurons (D), were shown, respectively. E–G) Representative fluorescent images of proAVP and HRD1 staining in PVN of *Atg7^AVP^* and wildtype control mice were shown (E), HRD1 staining intensities of AVP neurons and non‐PVN neurons (control) were quantified in *WT* (F) and *Atg7^AVP^* (G) mice, at indicated ages postnatally. Values, mean ± SEM. ^*^ *p* < 0.05; ^**^ *p* < 0.01; ^***^ *p* < 0.001 by *one‐way ANOVA* (B–D), and *two‐way ANOVA* (F‐G). Data represents at least 3 mice in each group. Each dot represents the staining intensity of one field (B‐G).

To confirm the mechanistic and physiological roles of autophagy in regulating ERAD activity in vivo, HRD1 protein levels and proAVP maturation efficiency were analyzed in autophagy or ERAD deficiency mice. Immunostaining of HRD1 and proAVP demonstrated that HRD1 protein levels in AVP neurons were significantly more abundant compared with adjacent non‐AVP neurons in the PVN (**Figure** **8**A,B). Consistently, HRD1 protein levels were significantly increased in *Atg7*‐deficient AVP neurons, while decreased with *Sel1L*‐deficiency as indicated previously (Figure 8C),^[^ ^24^ ^]^ while the numbers of proAVP positive neurons remained comparable among the three genotypes (Figure 8D).

To investigate the potential role of HRD1 elevation in the development of late‐onset diabetes insipidus phenotype in *Atg7^AVP^* mice, HRD1 protein levels and proAVP maturation efficiency were analyzed across the disease progression. *Atg7^AVP^* and control mice were analyzed at around 3 weeks, 4 months and 10 months postnatally, which represent after weaning, before, and after diagnosis of diabetes insipidus in *Atg7^AVP^* mice, respectively (Figure 8E). The results showed that beyond the enrichment of HRD1 protein levels in AVP neurons compared with the adjacent non‐PVN neurons (Figure 8F), HRD1 protein levels were continuously elevated with aging from 3 weeks to 10 months of age in the AVP neurons in WT mice (Figure 8E,F). Although HRD1 protein levels in AVP neurons of *Atg7^AVP^* mice were elevated compared with WT mice (Figure 8A–C), they failed to increase from 3 weeks to 4 months of age in AVP neurons (Figure 8G) and were even decreased compared with WT mice from 4 months to 10 months of age (Figure 8E–G). These data demonstrate the dynamic protein expression pattern of HRD1 in AVP neurons of *Atg7^AVP^* mice was associated with the onset of diabetes insipidus around 6 months of age and the continuous decline afterward (Figure 3G), which might contribute to the development of the late‐onset diabetes insipidus. These data also indicate that aging is associated with increased protein misfolding and less efficient clearance of misfolded proteins by ERAD and autophagy, which would finally lead to the degeneration of AVP neurons. Thus, either autophagy or ERAD deficiency in AVP neurons will result in impairment of misfolded proAVP clearance and diabetes insipidus.

### 2.9 Combined Deficiency of Autophagy and ERAD Leads to Exacerbated Diabetes Insipidus Phenotype and Loss of AVP Neurons

**Figure 9:**
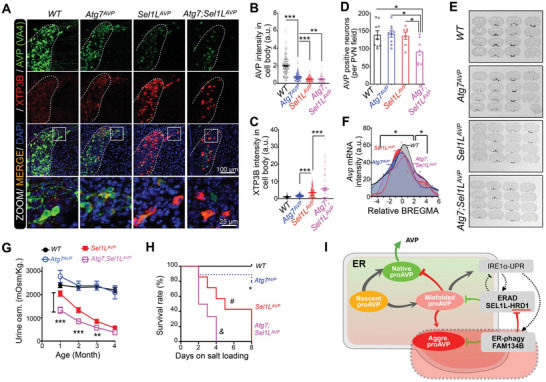
Combined deficiency of autophagy and ERAD leads to exacerbated diabetes insipidus phenotype and loss of AVP neurons. A‐D) Representative fluorescent images of proAVP and XTP3B in the PVNs of the indicated mice at 4 weeks of age, with zoomed images shown below (A). Quantifications of AVP intensity (B) and XTP3B intensity (C) in the cell bodies of AVP neurons from each genotype of mice were shown. The numbers of AVP‐positive neurons at indicated PVN regions are shown in (D). E,F) Representative images of ^35^S labeled RNA probe based in situ hybridization against proAVP mRNA in the PVNs of the indicated genotypes of mice. Images were shown as serious sectioning, and the mice were 9–15 weeks of age (E), with quantifications of AVP mRNA intensity shown as curves for each slice after autoradiography (F). G) The osmolality of freshly collected urine samples from each genotype at the indicated ages were analyzed. At least 5 mice from each group were included. H) Survival curve of mice with indicated genotypes fed on salty water for 8 days. Mice aged between 3–6 months were used, 4 to 8 mice in each group. I) Proposed model of the dual protection system for maintaining proAVP maturation and water homeostasis through autophagy and ERAD. Values, mean ± SEM. *p < 0.05; **p < 0.01; ***p < 0.001 by *one‐way ANOVA* (B‐D, F‐G). *, *p* < 0.05 between *WT* and *Atg7^AVP^* mice; #*p* < 0.05 between *Sel1L^AVP^* and *Atg7^AVP^* mice; &*p* < 0.05 between *Atg7;Sel1L^AVP^* and *Sel1L^AVP^* mice (H). Data represents at least 3 mice in each group. Each dot represents the staining intensity of one field (B–C).

To explain why autophagy deficiency in AVP neurons leads to late‐onset diabetes insipidus, we proposed a model that elevated HRD1 protein level with autophagy deficiency might be responsible for maintaining proAVP maturation efficiency by promoting the degradation of newly synthesized misfolded proteins before they formed aggregates, thus minimized the demand of autophagy activity. Therefore, ablation of ERAD activity in addition to autophagy deficiency would exaggerate the diabetes insipidus phenotype. To test the hypothesis and confirm the protective role of ERAD activation in proAVP maturation with autophagy deficiency in vivo, mice with compound deficiency of *Sel1L* and *Atg7* in AVP neurons were generated *(Atg7;Sel1L^AVP^* double deficient mice, referred to as *DKO*), which also led to *Hrd1* deficiency (**Figure** **8**C).^[^ ^24^, ^45^ ^]^ ProAVP protein level in the cell body of AVP neurons from *DKO* mice was reduced compared with *Atg7^AVP^* mice (**Figure** **9**A,B). XTP3B intensity was significantly increased in individual neurons of *DKO* mice compared with either *Atg7^AVP^* or *Sel1L^AVP^* mice (Figure 9C), indicating the elevated ER stress levels. Notably, AVP neuron numbers were reduced in *DKO* mice compared with either *Atg7^AVP^* or *Sel1L^AVP^* mice (Figure 9D). Further, the mRNA expression levels of proAVP in each genotype of mice were assessed by radioactive‐labeling based in situ hybridization in the PVN and SON regions of brain tissues. A significant reduction of AVP mRNA levels in *Sel1L^AVP^* and *DKO* mice was observed compared with WT mice (Figure 9E,F). To confirm the physiological role of ERAD activation with autophagy deficiency in water homeostasis, fresh urine osmolality of the 4 genotypes of mice were monitored from weaning to around 4 months of age. Our data showed that *DKO* mice exhibited a more severe impairment of water balance compared with either *Atg7^AVP^* or *Sel1L^AVP^* mice (Figure 9G). When mice were fed by salty water to induce osmotic stress, *Atg7^AVP^* mice showed increased susceptibility to osmotic stress‐induced death compared with WT mice, but less susceptible compared with *Sel1L^AVP^* mice (Figure 9H). Strikingly, *DKO* mice showed increased susceptibility to osmotic stress‐induced death compared with either *Atg7^AVP^* or *Sel1L^AVP^* mice (Figure 9H). Besides, our data show that both autophagy and ERAD play critical roles in maintaining proAVP maturation under physiological or pathological conditions. The combined deficiency of autophagy and ERAD results in synergistic damage to the critical proteostasis in AVP neurons as well as the neuronal survival. These data suggest that HRD1 elevation might mediate the adaptive response of autophagy deficiency in AVP neurons to facilitate the clearance of misfolded proAVP clearance and promote the mice survival under osmotic stress as proposed (Figure 9I).

## 3 Discussion

Accumulation of misfolded or aggregated proteins is a recognized pathogenic mechanism in various disease states including neurodegenerative diseases.^[^ ^46^ ^]^ However, how misfolded proteins accumulate and aggregate, as well as how they are cleared, in neurons under physiological or stress conditions in vivo remain largely unclear. The limited knowledge of ERQC mechanisms in neurons also limited the availability of drug targets and therapeutics against neurodegenerative diseases. In this study, we demonstrate that autophagy and ERAD coordinately prevent the accumulation of misfolded and aggregated proAVP to protect the endocrine neurons from degeneration under physiological and pathological conditions. We demonstrate that FAM134B mediated ER‐phagy is responsible for degrading ERAD‐resistant proAVP aggregates, and for regulating ERAD activity by degrading HRD1. We propose a sophisticated model on the ERQC mechanisms in endocrine neurons, that autophagy and ERAD form a dual‐protection system to safeguard the neuroendocrine functionality and degeneration by orchestrating the clearance of toxic misfolded proteins in the ER (Figure 9I).

Autophagy serves to protect the cells from cytotoxicity through degrading toxic protein aggregates, pathogens, and damaged organelles, which dysfunction in neurons leads to many diseases.^[^ ^47^ ^]^ The accumulation of misfolded proteins can activate autophagy or ERAD activities via UPR pathway activation.^[^ ^8^, ^9^, ^48^ ^]^ ERAD deficiency leads to the accumulation of misfolded proteins in the ER, such as proAVP, POMC (Proopiomelanocortin), proinsulin, and lipoprotein lipase.^[^ ^8^, ^21^, ^24^, ^32^, ^49^ ^]^ However, how misfolded or aggregated proteins in the ER are involved in neuron degeneration remains largely unknown. Through bioinformatic analysis of hypothalamic neurons during development and under dehydration, we identified the activation of ERAD and autophagy with increased neuroendocrine function, like AVP production, which are the two major ERQC pathways for protein degradation (Figure 1). The interactome analysis of WT proAVP in vitro by TurboID confirmed the involvement of ERAD and autophagy pathway in its protein quality control process (Figure 2). Unexpectedly, we demonstrated that *ATG7^AVP^* mice developed late‐onset diabetes insipidus under normal conditions (Figure 3), but showed overall reduction in proAVP synthesis with comparable maturation efficiency and increased ER stress in AVP neurons, before the onset of diabetes insipidus (Figure 4). Osmotic stress under salty water feeding increased proAVP synthesis and accelerated the development of diabetes insipidus in *ATG7^AVP^* mice (Figure 5). TurboID analysis of G57S FNDI mutant proAVP, which is misfolding‐ and aggregating‐prone, identified reduced ERAD components interacting with G57S mutant compared with WT proAVP (Figure 6). Biochemical analysis further demonstrated the FAM134B mediated ER‐phagy was responsible for the degradation of G57S mutant rather than WT proAVP under starvation‐induced activation of autophagy (Figure 6). Surprisingly, we noticed elevated HRD1 protein level in either ATG5 or ATG7 deficiency, and confirmed that FAM134B promoted the ER‐phagy‐dependent degradation of HRD1, while increased HRD1 promoted the clearance misfolded proAVP (Figure 7). The elevation of HRD1 protein level with autophagy deficiency was confirmed in AVP neurons of *ATG7^AVP^* mice, as well as during the aging process in AVP neurons of WT mice (Figure 8). Furthermore, we demonstrated that combined inhibition of ERAD and autophagy in AVP neurons led to even more severe accumulation of misfolded proAVP in vitro, and led to even most severe diabetes insipidus phenotype in vivo, compared with deficiency of either pathway (Figure 9). Finally, deficiency of both *Sel1L* and *Atg7* in AVP neurons in mice resulted in increased neuronal loss and vulnerability to osmotic stress‐induced death (Figure 9). Thus, we demonstrate that there might be an autoregulated, finely tuned ER proteostatic strategy to adaptively increase the function of either ERAD or autophagy pathway to minimize the accumulation of misfolded or aggregated prohormones to protect the endocrine neurons.^[^ ^8^, ^9^ ^]^

Although it is reported that FAM134B was responsible for initiating misfolded protein induced ER‐phagy,^[^ ^50^ ^]^ it is noteworthy to mention that FAM134B is involved in the degradation of both proAVP aggregates and endogenous HRD1 (Figures 6 and 7). This is for the first time, so far as we know, to demonstrate that autophagy could regulate ERAD activity by directly targeting HRD1 for degradation by FAM134B mediated ER‐phagy. Thus, we would also propose that FAM134B mediated ER‐phagy might degrade a subdomain of ER structure, which includes the co‐compartmentalized proAVP aggregates and/or ER‐resident ERAD components like HRD1. It remains unclear whether HRD1 or other ERAD components could be predisposed to binding with ER‐phagy receptor(s) like FAM134B for the fine‐tuning of ERQC process,^[^ ^38^ ^]^ which merits further study. Additionally, it remains to be clarified why SEL1L is not efficiently degraded by starvation‐induced autophagy, as seen in our experimental condition. We would propose the adaptive response mechanism of ERAD activation with autophagy deficiency as *Autophagy Deficiency Activated Protein Turnover in the ER* (ADAPTER), which could explain the autoregulatory mechanism of the ERQC system in mammalian cells to restore ER homeostasis under various stress conditions.

The ER is responsible for the synthesis of membranes for various vesicles as well as serves as a contact site for multiple organelles, including autophagosomes, and ER turnover is also regulated by other cellular processes.^[^ ^15^ ^]^ Thus, the protein quality control process in the ER could be affected by other organelles.^[^ ^51^, ^52^ ^]^ Reports showed that ER could be selectively removed through ER‐phagy receptors during intestine development,^[^ ^51^ ^]^ and selective autophagy of the ER also enabled the efficient degradation of specific proteins in the ER or ER subdomains by ER‐phagy receptors, including FAM134B, CCPG1, TEX264, ATL3, CALCOCO1, and CDK5RAP3.^[^ ^15^ ^]^ Another report showed that both RTN3 and BECLIN1, but not CCPG1, SEC62, or FAM134B, were involved in ER‐phagy dependent degradation of Akita‐mutant proinsulin aggregates. The C28F mutant POMC and G57S mutant proAVP were also degraded by RTN3‐mediated ER‐phagy in HEK293T cells in standard culture media without induction of autophagy.^[^ ^25^ ^]^ However, under EBSS treatment‐induced autophagy, we demonstrated through both gain and loss of function assays that FAM134B was involved in promoting the degradation of proAVP aggregates but not native proAVP in vitro (Figure 6). The difference in the dependence of ER‐phagy receptors in the two studies could be attributed to the different cell culture conditions.^[^ ^25^ ^]^ Additionally, ubiquitination of FAM134B by AMFR was demonstrated to facilitate FAM134B mediated dynamic flux of ER‐phagy,^[^ ^53^ ^]^ and FAM134B has been shown to be responsible for the lysosomal degradation of an ER‐localized disease mutant of alpha‐1‐antitrypsin (Z‐AAT) via its LIR motif.^[^ ^50^ ^]^ These reports indicate the clues for further studies on how the FAM134B determines its selectivity on various substrates, and how it is regulated, and how it binds to and degrades proAVP aggregates.

The physiological and cellular functions of autophagy have been intensively studied, and recent findings have shown increased interest in the roles of autophagy in maintaining ER homeostasis.^[^ ^8^, ^9^, ^21^, ^54^ ^]^ Different proteins or different folding statuses of a protein in the ER might be selectively degraded by ERAD or autophagy.^[^ ^20^ ^]^ We demonstrated that proAVP aggregates were resistant to degradation by the ERAD pathway, but were amenable to degradation by autophagy both in vitro and in vivo (Figures 6, 7, 8). Our results from either ERAD deficiency with inhibited autophagy, or autophagy deficiency with inhibited ERAD indicate, that both autophagy and ERAD are essential for maintaining proAVP maturation and water homeostasis (Figure 9). In this study, we showed that proAVP export efficiency was less affected in *Atg7*‐deficient AVP neurons compared with *Sel1L*‐deficiency. Indeed, the subcellular distribution of proAVP as well as markers of ER proteostasis were distinct between Sel1L‐deficient and *Atg7*‐deficient AVP neurons, which partially explains the differential dispensability and sensitivity to stress condition of each genotype in water homeostasis. Reduced proAVP in axons of *Atg7*‐deficient AVP neurons with late‐onset diabetes insipidus suggests that, autophagy acts as more of a safeguard or second‐line mechanism after ERAD in maintaining proAVP maturation when no proAVP aggregates were accumulated, while ERAD serves as a first‐line defense mechanism in preventing the accumulation of misfolded or aggregated proAVP. Future studies by ectopic expression of HRD1 in AVP neuron would provide more direct evidence on the protective role of elevated HRD1 expression against osmotic stress.

Combined deficiency of ERAD and autophagy in AVP neurons exacerbated the diabetes insipidus phenotype compared with either gene deficiency, and increased the sensitivity to osmotic stress‐induced animal death (Figure 9). Different cell types or tissues showed different vulnerability to various stresses with either ERAD or autophagy deficiency in vivo;^[^ ^8^, ^9^, ^21^, ^54^ ^]^ our data suggest a mechanism, at least for a subset of cell types, that ERAD could be activated with autophagy deficiency as a compensatory mechanism to reduce the risk of forming toxic protein aggregates (Figure 9I). However, in *Atg7^AVP^* mice under osmotic or aging stresses, the compensatory increase of ERAD component in AVP neurons might not be sufficient to combat the continuous demand of enhancing ERAD capacity, which finally could result in diabetes insipidus. Taken together, these data demonstrate the adaptive roles of either autophagy or ERAD in response to the deficiency of the other pathway, both of which could contribute to reducing the accumulation of misfolded or aggregated proteins and maintaining ER proteostasis and functionality. We propose to term this mechanism as a “Dual protection system of prohormone maturation in the ER (DuPPE)” for maintaining the maturation of proAVP, which might be extended to the understanding on the ERQC of other prohormones like proinsulin or POMC.^[^ ^9^, ^49^ ^]^ This mechanism also suggests a paradigm of an autoregulatory mechanism in the ER protein quality control for a subset of prohormones,^[^ ^55^ ^]^ wherein endocrine neurons adapt to increased protein synthesis and accumulation of wrongly folded proteins by activating either the ERAD or autophagy pathways or both, depending on the proteostatic status in the ER.

Previous studies reported that IRE1α was involved in the activation of autophagy,^[^ ^9^ ^]^ and that SEL1L‐HRD1 ERAD regulated IRE1α activation through promoting its degradation.^[^ ^44^ ^]^ This study further suggests an improved understanding on the cell type‐specific function of ERQC pathways in orchestrating the ER proteostasis on endocrine neurons, as well as their interplay in the pathogenesis of various protein‐misfolding related human diseases, such as endocrine neuron dysfunction, neurodegenerative diseases, and diabetes. Together with a recent report that ZIP7 (SLC39A7 in humans) prevents the neurodegeneration in Drosophila through enhancing ERAD activity against misfolded Rhodopsin,^[^ ^56^ ^]^ our data adds another layer of evidence on the protective role of ERAD in neurodegeneration in mice. Besides, activation of autophagy by rapamycin reduced FNDI mutant proAVP aggregates in AVP neurons of mice;^[^ ^26^ ^]^ However, whether and how UPR pathways are responsible for mediating the activation and the crosstalk between autophagy and ERAD pathways, as well as when and where to activate autophagy or ERAD pathways to reduce neurodegeneration in vivo, remains to be investigated. Therefore, promoting ERAD or autophagy functionality through selected chemicals or gene(s) delivery to target misfolded or aggregated pathogenic proteins for degradation could provide novel insights into treating protein folding diseases.

In summary, these findings underscore the importance of autophagy and ERAD pathways working in concert to achieve control of prohormone maturation, and identify a role for FAM134B mediated ER‐phagy in bridging the crosstalk between these ERQC pathways. A model of an auto‐regulated dual‐protection system was proposed in endocrine neurons for maintaining their physiological function by orchestrating the folding and degradation of peptide hormones. Thus, this study provides new insights into understanding the pathogenic mechanisms and therapeutic strategies against aberrant proteostasis‐related diseases.

## 4 Experimental Section

Supporting Information is available from the Wiley Online Library.

## Conflict of Interest

The authors declare no conflict of interest.

## Author Contributions

X.P., X.H., S.W., and N.X. contributed equally to this work. L.Q., G.S., and J.C. initiated and designed the project. G.S., X.P., X.H., S.W., and X.H. designed and performed most of the experiments and edited the manuscript. N.X. performed the single cell and bulk RNA‐seq analysis. H.W., D.S., and J.Y. performed part of the experiments and provided technical assistance. M.S., H.W., and C.L. provided critical comments and edited the manuscript. S.L. and W.M. provided critical support for the bioinformatic analysis and TurboID assays. Y.C. provided funding support and critical comments. G.S. and L.Q. wrote the original manuscript and provided funding support. X.P., J.C., and D.S. provided critical comments and edited the manuscript.

## Supporting information



Supporting Information

## Data Availability

The data that support the findings of this study are available from the corresponding author upon reasonable request.;

## References

[1] A. Zbinden , M. Perez‐Berlanga , P. De Rossi , M. Polymenidou , P. Separation , Dev. Cell 2020, 55, 45. 33049211 10.1016/j.devcel.2020.09.014

[2] J. Lim , Z. Yue , Dev. Cell 2015, 32, 491. 25710535 10.1016/j.devcel.2015.02.002 PMC4376477

[3] M. F. Princiotta , D. Finzi , S. B. Qian , J. Gibbs , S. Schuchmann , F. Buttgereit , J. R. Bennink , J. W. Yewdell , Immunity 2003, 18, 343. 12648452 10.1016/s1074-7613(03)00051-7

[4] A. E. Frakes , A. Dillin , Mol. Cell 2017, 66, 761. 28622521 10.1016/j.molcel.2017.05.031

[5] C. Hetz , K. Zhang , R. J. Kaufman , Nat. Rev. Mol. Cell Biol. 2020, 21, 421. 32457508 10.1038/s41580-020-0250-z PMC8867924

[6] L. Qi , B. Tsai , P. Arvan , Trends Cell Biol. 2017, 27, 430. 28131647 10.1016/j.tcb.2016.12.002 PMC5440201

[7] C. J. Guerriero , J. L. Brodsky , Physiol. Rev. 2012, 92, 537. 22535891 10.1152/physrev.00027.2011 PMC4162396

[8] S. A. Wu , C. Shen , X. Wei , X. Zhang , S. Wang , X. Chen , M. Torres , Y. Lu , L. L. Lin , H. H. Wang , A. H. Hunter , D. Fang , S. Sun , M. I. Ivanova , Y. Lin , L. Qi , Nat. Commun. 2023, 14, 3132. 37253728 10.1038/s41467-023-38690-4 PMC10229581

[9] N. Shrestha , M. Torres , J. Zhang , Y. Lu , L. Haataja , R. B. Reinert , J. Knupp , Y. J. Chen , G. Parlakgul , A. P. Arruda , B. Tsai , P. Arvan , L. Qi , J. Clin. Invest. 2023, 133, e163584. 36346671 10.1172/JCI163584 PMC9797341

[10] S. Yoshida , X. Wei , G. Zhang , C. L. O'Connor , M. Torres , Z. Zhou , L. Lin , R. Menon , X. Xu , W. Zheng , Y. Xiong , E. Otto , C. A. Tang , R. Hua , R. Verma , H. Mori , Y. Zhang , C. A. Hu , M. Liu , P. Garg , J. B. Hodgin , S. Sun , M. Bitzer , L. Qi , J. Clin. Invest. 2021, 131, e143988. 33591954 10.1172/JCI143988 PMC8011890

[11] Z. Zhou , M. Torres , H. Sha , C. J. Halbrook , F. Van den Bergh , R. B. Reinert , T. Yamada , S. Wang , Y. Luo , A. H. Hunter , C. Wang , T. H. Sanderson , M. Liu , A. Taylor , H. Sesaki , C. A. Lyssiotis , J. Wu , S. Kersten , D. A. Beard , L. Qi , Science 2020, 368, 54. 32193362 10.1126/science.aay2494 PMC7409365

[12] A. Bhattacharya , L. Qi , J. Cell Sci. 2019, 132, jcs232850. 31792042 10.1242/jcs.232850 PMC6918741

[13] R. Y. Hampton , R. G. Gardner , J. Rine , Mol. Biol. Cell 1996, 7, 2029. 8970163 10.1091/mbc.7.12.2029 PMC276048

[14] B. Mueller , E. J. Klemm , E. Spooner , J. H. Claessen , H. L. Ploegh , Proc. Natl. Acad. Sci. USA 2008, 105, 12325. 18711132 10.1073/pnas.0805371105 PMC2527910

[15] K. Mochida , H. Nakatogawa , EMBO Rep. 2022, 23, e55192. 35758175 10.15252/embr.202255192 PMC9346472

[16] A. Khaminets , T. Heinrich , M. Mari , P. Grumati , A. K. Huebner , M. Akutsu , L. Liebmann , A. Stolz , S. Nietzsche , N. Koch , M. Mauthe , I. Katona , B. Qualmann , J. Weis , F. Reggiori , I. Kurth , C. A. Hubner , I. Dikic , Nature 2015, 522, 354. 26040720 10.1038/nature14498

[17] T. Hidvegi , M. Ewing , P. Hale , C. Dippold , C. Beckett , C. Kemp , N. Maurice , A. Mukherjee , C. Goldbach , S. Watkins , G. Michalopoulos , D. H. Perlmutter , Science 2010, 329, 229. 20522742 10.1126/science.1190354

[18] B. Levine , G. Kroemer , Cell 2019, 176, 11. 30633901 10.1016/j.cell.2018.09.048 PMC6347410

[19] M. Molinari , Dev. Cell 2021, 56, 949. 33765438 10.1016/j.devcel.2021.03.005

[20] S. A. Houck , H. Y. Ren , V. J. Madden , J. N. Bonner , M. P. Conlin , J. A. Janovick , P. M. Conn , D. M. Cyr , Mol. Cell 2014, 54, 166. 24685158 10.1016/j.molcel.2014.02.025 PMC4070183

[21] X. Zhang , C. Young , X. H. Liao , S. Refetoff , M. Torres , Y. Tomer , M. Stefan‐Lifshitz , H. Zhang , D. Larkin , D. Fang , L. Qi , P. Arvan , JCI Insight 2023, 8, e169937. 37345654 10.1172/jci.insight.169937 PMC10371246

[22] R. L. Wiseman , J. S. Mesgarzadeh , L. M. Hendershot , Mol. Cell 2022, 82, 1477. 35452616 10.1016/j.molcel.2022.03.025 PMC9038009

[23] L. Plate , R. L. Wiseman , Trends Cell Biol. 2017, 27, 722. 28647092 10.1016/j.tcb.2017.05.006 PMC5612838

[24] G. Shi , D. Somlo , G. H. Kim , C. Prescianotto‐Baschong , S. Sun , N. Beuret , Q. Long , J. Rutishauser , P. Arvan , M. Spiess , L. Qi , J. Clin. Invest. 2017, 127, 3897. 28920920 10.1172/JCI94771 PMC5617659

[25] C. N. Cunningham , J. M. Williams , J. Knupp , A. Arunagiri , P. Arvan , B. Tsai , Mol. Cell 2019, 75, 442. 31176671 10.1016/j.molcel.2019.05.011 PMC6688957

[26] T. Miyata , D. Hagiwara , Y. Hodai , T. Miwata , Y. Kawaguchi , J. Kurimoto , H. Ozaki , K. Mitsumoto , H. Takagi , H. Suga , T. Kobayashi , M. Sugiyama , T. Onoue , Y. Ito , S. Iwama , R. Banno , M. Matsumoto , N. Kawakami , N. Ohno , H. Sakamoto , H. Arima , iScience 2020, 23, 101648. 33103081 10.1016/j.isci.2020.101648 PMC7578753

[27] S. Gaspari , G. Labouebe , A. Picard , X. Berney , A. R. Sanchez‐Archidona , B. Thorens , EMBO Rep. 2023, 24, e57344. 37314252 10.15252/embr.202357344 PMC10398655

[28] C. Atila , J. Refardt , M. Christ‐Crain , Nat. Rev. Endocrinol. 2024, 20, 487. 38719970 10.1038/s41574-024-00998-6

[29] L. K. Hansen , S. Rittig , G. L. Robertson , Trends Endocrinol. Metab. 1997, 8, 363. 18406826 10.1016/s1043-2760(97)00157-4

[30] J. Birk , M. A. Friberg , C. Prescianotto‐Baschong , M. Spiess , J. Rutishauser , J. Cell Sci. 2009, 122, 3994. 19825939 10.1242/jcs.051136

[31] M. A. Friberg , M. Spiess , J. Rutishauser , J. Biol. Chem. 2004, 279, 19441. 14996841 10.1074/jbc.M310249200

[32] D. Hagiwara , H. Arima , Y. Morishita , L. Wenjun , Y. Azuma , Y. Ito , H. Suga , M. Goto , R. Banno , Y. Sugimura , A. Shiota , N. Asai , M. Takahashi , Y. Oiso , Cell Death Dis. 2014, 5, e1148. 24675466 10.1038/cddis.2014.124 PMC3973212

[33] T. A. Russell , M. Ito , M. Ito , R. N. Yu , F. A. Martinson , J. Weiss , J. L. Jameson , J. Clin. Invest. 2003, 112, 1697. 14660745 10.1172/JCI18616 PMC281642

[34] B. Chen , T. Ge , M. Jian , L. Chen , Z. Fang , Z. He , C. Huang , Y. An , S. Yin , Y. Xiong , J. Zhang , R. Li , M. Ye , Y. Li , F. Liu , W. Ma , Z. Songyang , Nat. Cell Biol. 2023, 25, 1004. 37322289 10.1038/s41556-023-01165-1

[35] T. C. Branon , J. A. Bosch , A. D. Sanchez , N. D. Udeshi , T. Svinkina , S. A. Carr , J. L. Feldman , N. Perrimon , A. Y. Ting , Nat. Biotechnol. 2018, 36, 880. 30125270 10.1038/nbt.4201 PMC6126969

[36] R. A. Romanov , E. O. Tretiakov , M. E. Kastriti , M. Zupancic , M. Haring , S. Korchynska , K. Popadin , M. Benevento , P. Rebernik , F. Lallemend , K. Nishimori , F. Clotman , W. D. Andrews , J. G. Parnavelas , M. Farlik , C. Bock , I. Adameyko , T. Hokfelt , E. Keimpema , T. Harkany , Nature 2020, 582, 246. 32499648 10.1038/s41586-020-2266-0 PMC7292733

[37] A. G. Pauza , A. S. Mecawi , A. Paterson , C. C. T. Hindmarch , M. Greenwood , D. Murphy , M. P. Greenwood , J. Neuroendocrinol. 2021, 33, e13007. 34297454 10.1111/jne.13007

[38] F. Reggiori , M. Molinari , Physiol. Rev. 2022, 102, 1393. 35188422 10.1152/physrev.00038.2021 PMC9126229

[39] J. Zhang , W. Zeng , Y. Han , W. R. Lee , J. Liou , Y. Jiang , Mol. Cell 2023, 83, 2524. 37390818 10.1016/j.molcel.2023.06.004 PMC10528928

[40] M. Komatsu , S. Waguri , T. Ueno , J. Iwata , S. Murata , I. Tanida , J. Ezaki , N. Mizushima , Y. Ohsumi , Y. Uchiyama , E. Kominami , K. Tanaka , T. Chiba , J. Cell Biol. 2005, 169, 425. 15866887 10.1083/jcb.200412022 PMC2171928

[41] M. Komatsu , Q. J. Wang , G. R. Holstein , V. L. Friedrich Jr , J. Iwata , E. Kominami , B. T. Chait , K. Tanaka , Z. Yue , Proc. Natl. Acad. Sci. USA 2007, 104, 14489. 17726112 10.1073/pnas.0701311104 PMC1964831

[42] S. Ma , A. E. Dubin , Y. Zhang , S. A. R. Mousavi , Y. Wang , A. M. Coombs , M. Loud , I. Andolfo , A. Patapoutian , Cell 2021, 184, 969. 33571427 10.1016/j.cell.2021.01.024 PMC7927959

[43] L. L. Lin , H. H. Wang , B. Pederson , X. Wei , M. Torres , Y. Lu , Z. J. Li , X. Liu , H. Mao , H. Wang , L. E. Zhou , Z. Zhao , S. Sun , L. Qi , Nat. Commun. 2024, 15, 1440. 38365914 10.1038/s41467-024-45633-0 PMC10873344

[44] S. Sun , G. Shi , H. Sha , Y. Ji , X. Han , X. Shu , H. Ma , T. Inoue , B. Gao , H. Kim , P. Bu , R. D. Guber , X. Shen , A. H. Lee , T. Iwawaki , A. W. Paton , J. C. Paton , D. Fang , B. Tsai , J. R. Yates Iii , H. Wu , S. Kersten , Q. Long , G. E. Duhamel , K. W. Simpson , L. Qi , Nat. Cell Biol. 2015, 17, 1546. 26551274 10.1038/ncb3266 PMC4670240

[45] S. Sun , G. Shi , X. Han , A. B. Francisco , Y. Ji , N. Mendonca , X. Liu , J. W. Locasale , K. W. Simpson , G. E. Duhamel , S. Kersten , J. R. Yates, 3rd, Q. Long , L. Qi , Proc. Natl. Acad. Sci. USA 2014, 111, E582. 24453213 10.1073/pnas.1318114111 PMC3918815

[46] W. E. Balch , R. I. Morimoto , A. Dillin , J. W. Kelly , Science 2008, 319, 916. 18276881 10.1126/science.1141448

[47] J. J. Collier , C. Guissart , M. Olahova , S. Sasorith , F. Piron‐Prunier , F. Suomi , D. Zhang , N. Martinez‐Lopez , N. Leboucq , A. Bahr , S. Azzarello‐Burri , S. Reich , L. Schols , T. M. Polvikoski , P. Meyer , L. Larrieu , A. M. Schaefer , H. S. Alsaif , S. Alyamani , S. Zuchner , I. A. Barbosa , C. Deshpande , A. Pyle , A. Rauch , M. Synofzik , F. S. Alkuraya , F. Rivier , M. Ryten , R. McFarland , A. Delahodde , et al., N. Engl. J. Med. 2021, 384, 2406. 34161705 10.1056/NEJMoa1915722 PMC7611730

[48] D. Senft , Z. A. Ronai , Trends Biochem. Sci. 2015, 40, 141. 25656104 10.1016/j.tibs.2015.01.002 PMC4340752

[49] G. H. Kim , G. Shi , D. R. Somlo , L. Haataja , S. Song , Q. Long , E. A. Nillni , M. J. Low , P. Arvan , M. G. Myers Jr , L. Qi , J. Clin. Invest. 2018, 128, 1125. 29457782 10.1172/JCI96420 PMC5824855

[50] Y. Sun , X.e. Wang , X. Yang , L. Wang , J. Ding , C.‐c. Wang , H. Zhang , X. Wang , Dev. Cell 2023, 58, 2761. 37922908 10.1016/j.devcel.2023.10.007

[51] R. Wang , T. M. Fortier , F. Chai , G. Miao , J. L. Shen , L. J. Restrepo , J. J. DiGiacomo , P. D. Velentzas , E. H. Baehrecke , Cell 2023, 186, 4172. 37633267 10.1016/j.cell.2023.08.008 PMC10530463

[52] S. A. Tooze , T. Yoshimori , Nat. Cell Biol. 2010, 12, 831. 20811355 10.1038/ncb0910-831

[53] A. Gonzalez , A. Covarrubias‐Pinto , R. M. Bhaskara , M. Glogger , S. K. Kuncha , A. Xavier , E. Seemann , M. Misra , M. E. Hoffmann , B. Brauning , A. Balakrishnan , B. Qualmann , V. Dotsch , B. A. Schulman , M. M. Kessels , C. A. Hubner , M. Heilemann , G. Hummer , I. Dikic , Nature 2023, 618, 394. 37225996 10.1038/s41586-023-06089-2 PMC10247366

[54] N. Shrestha , T. Liu , Y. Ji , R. B. Reinert , M. Torres , X. Li , M. Zhang , C. A. Tang , C. A. Hu , C. Liu , A. Naji , M. Liu , J. D. Lin , S. Kersten , P. Arvan , L. Qi , J. Clin. Invest. 2020, 130, 3499. 32182217 10.1172/JCI134874 PMC7324191

[55] Y. Liu , S. Liu , A. Tomar , F. S. Yen , G. Unlu , N. Ropek , R. A. Weber , Y. Wang , A. Khan , M. Gad , J. Peng , E. Terzi , H. Alwaseem , A. E. Pagano , S. Heissel , H. Molina , B. Allwein , T. C. Kenny , R. L. Possemato , L. Zhao , R. K. Hite , E. V. Vinogradova , S. S. Mansy , K. Birsoy , Science 2023, 382, 820. 37917749 10.1126/science.adf4154 PMC11170550

[56] X. Guo , M. Mutch , A. Y. Torres , M. Nano , N. Rauth , J. Harwood , D. McDonald , Z. Chen , C. Montell , W. Dai , D. J. Montell , Dev. Cell 2024, 59, 1655. 38670102 10.1016/j.devcel.2024.04.003 PMC11233247

