# Supplementary material for: Coordinated Role of Autophagy and ERAD in Maintaining Neuroendocrine Function by Preventing Prohormone Aggregation
Source: Adv Sci (Weinh). 2025 Mar 7;12(23):2411662. doi: 10.1002/advs.202411662 (PMC12199385; doi:10.1002/advs.202411662)
Supplement: Supplementary file 1 — Supporting Information [file ADVS-12-2411662-s001.docx]

# SUPPLEMENTARY FIGURES


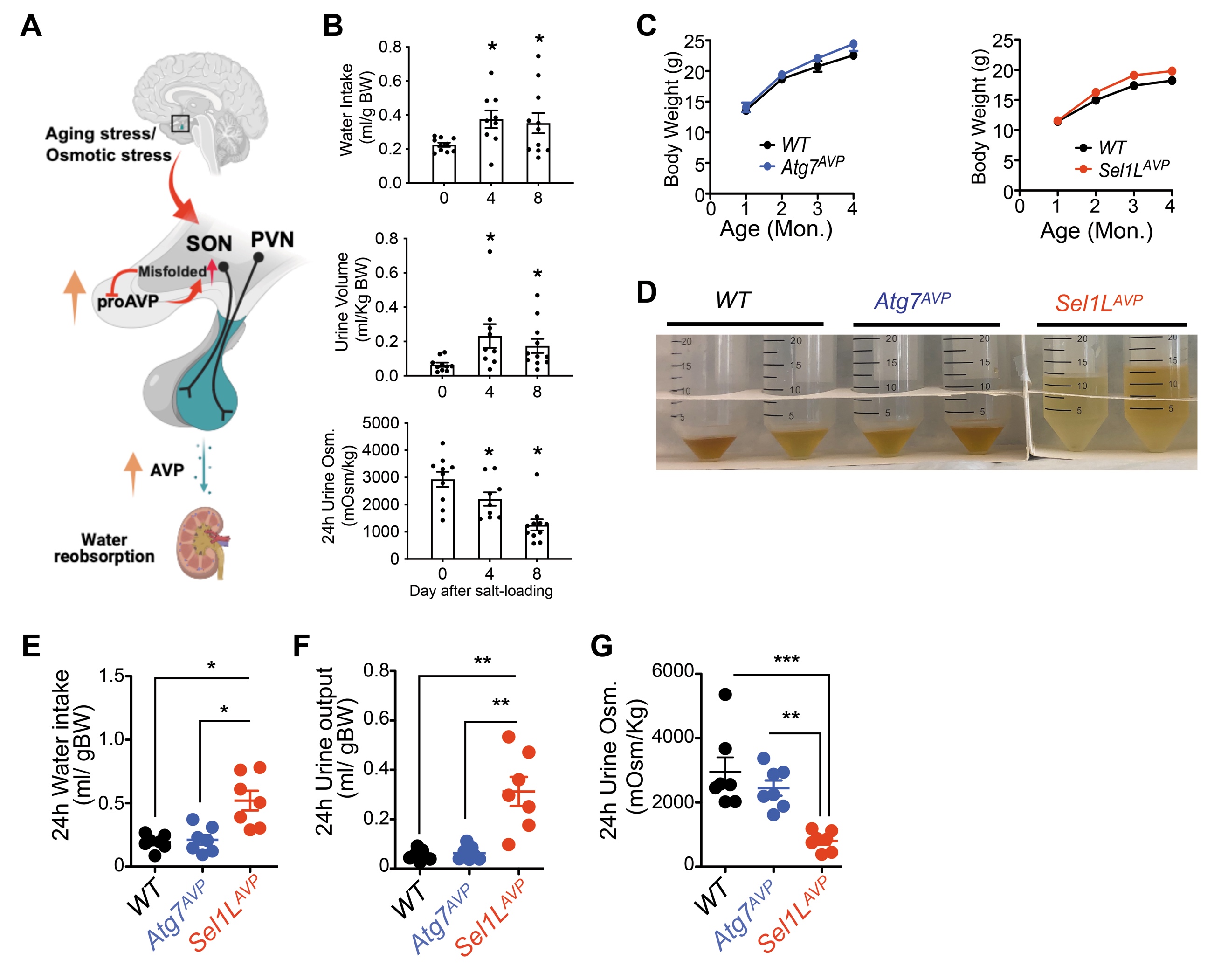


**Supplementary Figure S1. Autophagy deficiency in AVP neurons shows intact water homeostasis in young mice.**

**(A-B)** A cartoon model demonstrating AVP production in response to osmotic stress (A). Osmotic stress was induced by 2% NaCl water feeding, showing increased water intake, increased 24h urine volume, and decreased 24h urine osmolality (B).

**(C)** Body weight curves of *Atg7^AVP^*, *Sel1L^AVP^*, and *WT* control mice.

**(D)** Representative images of 24h urine volumes of *Atg7^AVP^*, *Sel1L^AVP^*, and *WT* mice as indicated**.**

**(E-G)** 24h water intake, 24h urine volumes, and urine osmolality (24h) of *Atg7^AVP^*, *Sel1L^AVP^*, and *WT* control mice at ages between 7-10 weeks.

Values, mean ± SEM. *, *p*<0.05; **, *p*<0.01; ***, *p*<0.001 by *Student’s t-tests* (B) *or one-way* ANOVA (E-G).


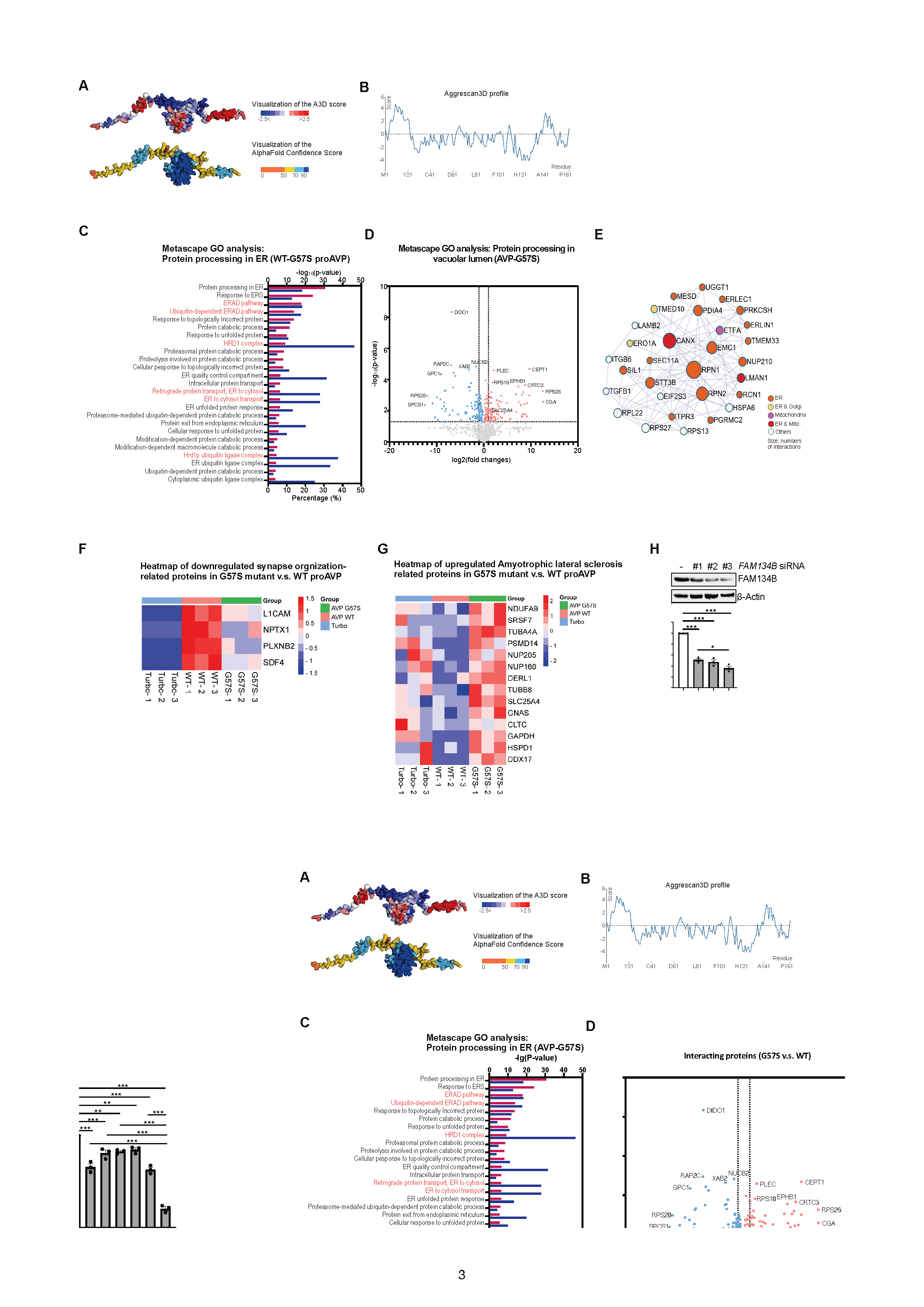


**Supplementary Figure S2. Predicted proAVP aggregation propensity and TurboID analysis of G57S mutant proAVP.**

**(A-B)** Predicted proAVP aggregation propensity by showing the hydrophobic domains (Red) in two ends that could form amyloid-like fibrillar aggregates when misfolded, with the AlphaFold confidence score shown below, respectively (A). The quantification of the aggregation score was shown as well accordingly (B).

**(C)** GO analysis of G57S mutant proAVP enriched preys functioning in protein processing in ER.

**(D)** Volcano plot for analyzing the different interacting proteins comparing with G57S mutant and WT proAVP identified through TurboID.

**(E)** Network of enriched proteins from G57S mutant proAVP by TurboID assay.

**(F-G)** Heatmap of downregulated ‘synapse organization-related proteins’ (F), and ‘upregulated Amyotrophic lateral sclerosis’ related proteins in G57S mutant proAVP.

**(H)** Western blot analysis of FAM134B protein levels with differential siRNAs. Values, mean ± SEM. *, *p*<0.05; ***, *p*<0.001 by *one-way* ANOVA.


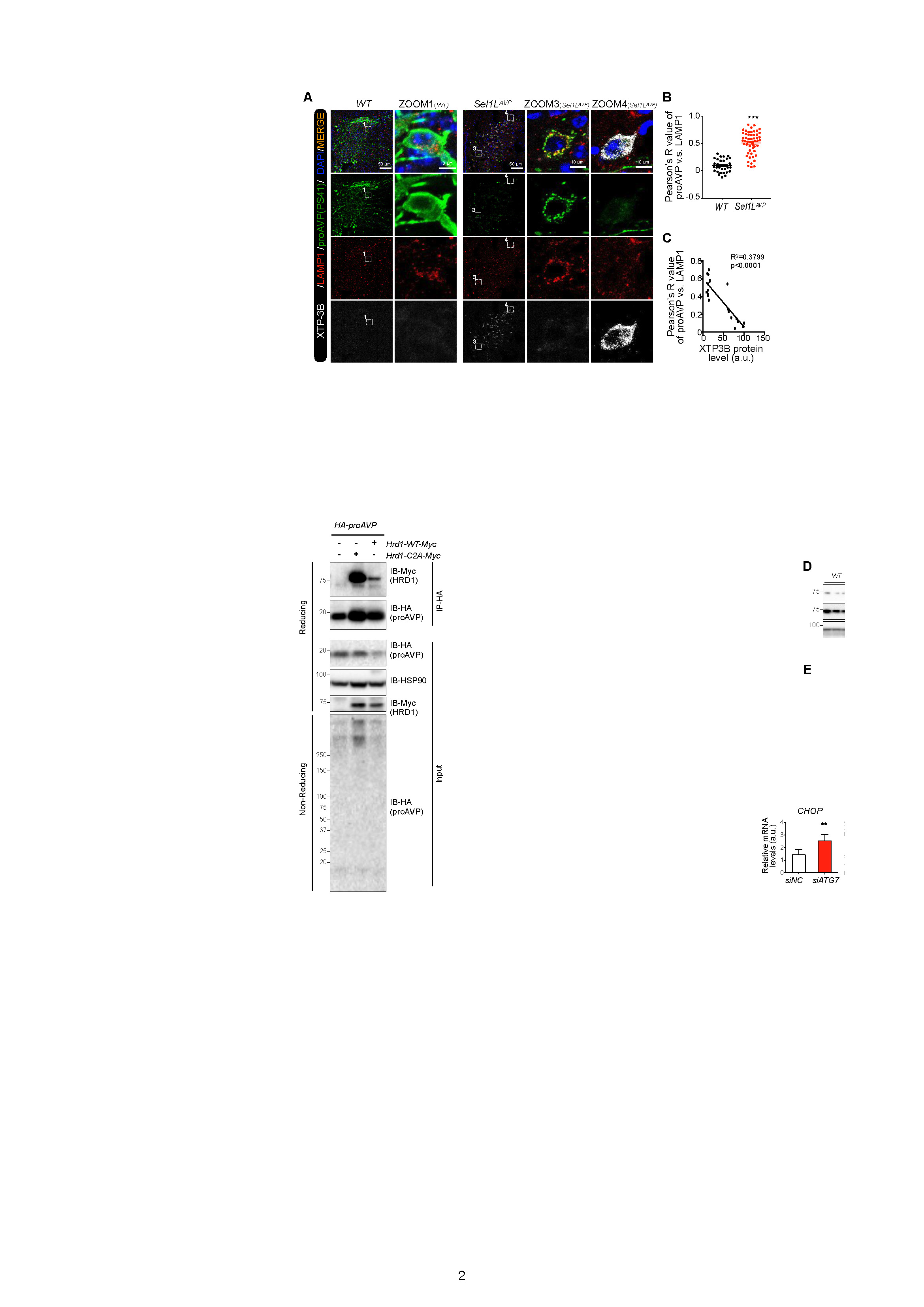


**Supplementary Figure S3. The crosstalk between autophagy and ERAD pathways in response to the deficiency of either autophagy or ERAD pathway.**

**(A-C)** Representative immunostaining images of proAVP, LAMP1, and XTP3B in PVNs of WT and *Sel1L^AVP^* mice fed regular water (A). Protein colocalization efficiency of proAVP and LAMP1 in individual AVP neurons of *WT* and *Sel1L^AVP^* mice (B), and the correlation between the colocalization efficiency and XTP3B protein expression levels in individual neurons of *Sel1L^AVP^* mice were quantified (C).

Mice analyzed here were fed ad libitum aged at around 4 months, n=2 mice each group. Values, mean ± SEM. ***, *p*<0.001 by *Student’s t-test*.

**MATERIALS AND METHODS**

**Bioinformatic analysis of RNA-seq data**

Neuronal clusters were labeled via metadata derived from GSE132730 [1], and further analyzed for AVP-positive cells through unsupervised clustering at a resolution of 0.2. The resulting UMAP visualizations (**Fig. 1A**) articulate the distribution of Avp mRNA expression within the various clusters. Advancing our analysis with Seurat [2] (version 4.3.0.1), we employed the AddModuleScore function to enrich our dataset for pathway activity pertaining to both 'Autophagy' and 'Response to ERS', which were downloaded from the GO database. This scoring system facilitated a detailed and quantifiable examination of pathway involvement across individual cells. DEGs were filtered using FindAllMarkers, and the fold change enrichment was calculated from the enrichment analysis via GeneRatio/BgRatio, executed using clusterProfiler (**Fig.1G**). For bulk RNA-seq data GSE175461 [3], genes exhibiting an absolute log2 fold change (|log2FC|) greater than 1 with an adjusted p-value of less than 0.05 were classified as differentially expressed genes (DEGs). These differentially expressed genes served as the input for subsequent enrichment analysis. The exhaustive analysis of enriched biological processes was conducted leveraging the ClusterProfiler package^[4]^ (version 4.6.2) in R. Furthermore, standardized matrices were employed to derive average gene expression levels from the enrichment outcomes in the 'Autophagy' and 'Response to ERS' RNA-seq pathways, illuminating their expression intensities and providing deeper insight into pathway dynamics.

**TurboID analysis for proAVP interacting proteins**

For stable cell lines, low-passage HeLa cells were purchased from ATCC and cultured in DMEM high glucose medium (Gibco) supplemented with 10% fetal bovine serum (FBS) (Excel Biosciences) at 37 °C with 5% CO_2_. Gene expressing lentiviral vectors were transfected into HEK293T cells together with packaging plasmid (pxPAX2) and envelope plasmid (pMD2.G) using Polyethylenimine (PEI, Polyscience, 24765-1). After 48 h, viruses were collected, filtered, and used to infect HeLa cells at low titer in the presence of 8 μg/mL polybrene for 6-8 h. The infected cells were selected by 2 μg/ml puromycin (Thermo, A11138-03) for 3 days. Stable cell lines were generated through infection with lentiviruses encoding the desired proteins followed by antibiotic selection as appropriate.

For TurboID labeling and MS sample preparation, TurboID was carried out as described with minor modifications [5]. Briefly, HeLa cells were pre-cultured in dialyzed serum (LONSERA) for 3 days. For large-scale affinity purification using stable expression cell lines, cells were seeded in 15-cm dishes at ~80% confluence (~2×10^7^ cells) and cultured with biotin (#B4639, Sigma) (50 µM) for biotinylation labeling. Cells were lysed with RIPA buffer (10 mM Tris-Cl pH 7.4, 140 mM NaCl, 0.1% SDS, 1 mM EDTA, 0.5 mM EGTA, 0.1% sodium deoxycholate, 1% Triton X-100, protease inhibitors cocktail, RNase) and the lysate was sonicated at 30% Amplification for 30 cycles of 2 seconds on and 8 seconds off (Sonics Vibra-Cell; VCX130PB) and centrifuged at 12,000 g for 10 min at 4℃. The supernatant was then incubated with 30 μL of streptavidin magnetic beads (Thermo, 88817) with rotation for 2 h at 4℃. Beads were sequentially washed and reduced in 20 mM DTT in 50 mM NH_4_HCO_3_ buffer containing 0.1% SDS for 45 min, then alkylated by adding IAA (final conc. 40 mM) for 30 min. The beads were washed with 50 mM NH_4_HCO_3_ for three times, followed by trypsin digestion overnight at 37℃. PireceTM C18 Tips (Thermo, cat# 87784) were used for peptide desalting according to the manufacturer instructions. LC-MS/MS was performed on an Ultimate 3000 RSLC nano system coupled with an Orbitrap Fusion Lumos Tribrid mass spectrometer (Thermo Fisher Scientific).

For MS data screening and analysis, Raw MS files (Thermo.raw format) were analyzed using MaxQuant (version 1.6.2.10) and searched against human UniProtKB/Swiss-Prot database. Three replications were analyzed for each analysis using “match between runs”. Parameters and preliminary data screening were as previously described [6]. Newly created proteinGroups files were filtered and calculated into prey fraction of total (FOT) values of each sample to represent the normalized abundance for facilitating comparison among baits. For screening enriched proximal interactors, only preys detected at least 2 unique peptides and with ≥ 3 folds enrichment compared with control were selected. Protein complexes hit by each group were analyzed by Metascape (https://metascape.org/gp/index.html#/main/) and selected CORUM when performing enrichment analysis. To construct GO barcodes, “GO Biological Processes” categories were applied for enrichment analysis, and GO terms with q value < 0.01 were classified into groups representing different biological processes. Interaction networks for bait-interactor or NC-protein complex were all visualized using Cytoscape v.3.8.2 (https://www.cytoscape.org/). The chosen volcano results were visualized using GraphPad Prism 8 and the R package pheatmap.

**Animal models**

The *Sel1L^fl/fl^*, *Atg7^fl/fl^* and *Sel1L^AVP^* mice were described previously [7, 8]. For generating AVP neuron specific Atg7-deficient mice (*Atg7^AVP^*) and Atg7-Sel1L dual-deficient mice (*Atg7;Sel1L^AVP^*), *Atg7^fl/fl^* or crossed *Sel1L;Atg7^fl/fl^* mice were crossed with mice expressing AVP promoter-driven *Cre* on the C57BL/6J background (JAX 023530,B6.CgAVP^tm1.1/(cre)Hze^/J; The Jackson Laboratory). *Cre* negative *Atg7^fl/fl^* or *Sel1L;Atg7^fl/fl^* mice were used as control mice. Mice were fed a chow diet (13% fat, 67% carbohydrate, and 20% protein, 2914; Harlan Teklad), and housed in a 12-hour light/12-hour dark cycle and under controlled temperature (20℃-22℃) in the mouse facility. All animal procedures were approved and done in accordance with the IACUC at the University of Michigan Medical School (PRO00010658).

**Water intake and urine output analysis and salt loading assays**

Mice were fed with 2% weight/volume NaCl salty water ad libitum with food for 4 to 8 consecutive days in metabolic cages (Techniplast USA Single Mouse Cage, 3600M021),with daily monitoring of water intake, food intake, and body weight. Mice were then sacrificed immediately. Mice were examined throughout each experiment for signs of dehydration, which would necessitate an immediate end to the experiment. Water intake and urine output analysis were conducted in metabolic cages (Techniplast, USA). Mice were housed individually for 24 hours with ad libitum access to food and water. Each session was separated by 48 hours. Food, water, and mouse body weight were measured before and after each session. Urine samples obtained from collecting tubes in metabolic cages (for 24 hours) or by bladder massage (fresh urine) were pipetted into microcentrifuge tubes and stored on ice. Samples were analyzed immediately for osmolality using a freezing point micro-osmometer (Fiske Model 210, Single Sample Micro Osmometer). Collected blood samples were kept at room temp for 10-15 minutes and then placed on ice and were later spun down (3,000 g for 15 minutes at 4°C) to separate serum and stored at -80°C before use.

**Preparation of brain sections and staining**

For immunofluorescence or *in situ* hybridization, mice were anesthetized by avertin tribromoethanol (Sigma-Aldrich, T4 840-2) or isoflurane, and then euthanized by transcardial fix-perfusion with 4% paraformaldehyde (PFA, Electron Microscopy Sciences, 19210). The brains were then fixed in 4% PFA for 2 hours at 4°C, dehydrated in 15% sucrose overnight at 4°C, and sectioned on a cryostat (Thermo Fisher Scientific Microm HM 550). Brain sections (30 μm) were stored in DEPC-containing anti-freezing media (50% 0.05M Sodium Phosphate pH=7.3, 30% Ethylene Glycol, 20% Glycerol) at −20°C. For immunostaining, free-floating brain sections were permeabilized in 0.3% Triton X-100 for 10 minutes at room temperature and then incubated in blocking solution (1% donkey serum and 0.03% Triton X-100 in 0.0.5M potassium phosphate-buffered saline [K-PBS]) for 30 minutes at room temperature. After blocking, free-floating brain sections were simultaneously incubated overnight with primary antibodies at 4°C, followed by 3 washes with K-PBST (0.03% Triton X-100 in 0.05 M K-PBS), and were incubated with secondary antibodies for 2 hours at room temperature. Brain sections were then mounted on gelatin-coated slides (SLD01-CS; Southern Biotech). Counterstaining and counting were performed with mounting medium containing DAPI (Vector H-1200) and a Fisherfinest Premium Coverslip (12-548-5P; Thermo Fisher Scientific). The colocalization of fluorophores was quantitatively assessed by drawing regions around individual cells, and the Pearson’s correlation co-efficiency was computed by using the ImageJ plugin, Coloc 2 (https://imagej.net/Coloc_2). Linear analysis between the colocalization of AVP vs. LAMP1 or XTP3B protein levels was assessed by measuring the Pearson’s R value as well as the indicated fluorophore intensity in individual AVP neuron as mentioned above, and then performed by Prism7. For single in situ hybridization (ISH), the single ISH was performed to determine the expression levels of Avp mRNA in the hypothalamic paraventricular nucleus (PVH). Briefly, a serious brain sections (3 mice in each genotypes) were mounted onto SuperFrost plus slides (Thermo Fisher Scientific), fixed in 10% NBF for 20 minutes, and cleared with xylene for 15 minutes. Slices were boiled in sodium citrate buffer (pH 6.0) for 10 minutes. The *Avp* DNA template was generated from mouse hypothalamic RNA by PCR amplification of a 188 bp region (NM_009732) containing the glycosylation site using follow pair of primers forward: ATCTGCTGCAGCGACGAGAG and reverse: CAGTAGACCCGGGGCTTGGCAGAA. Primers were acquired from IDT. The antisense radio-labeled ^35^S-Avp riboprobe was generated through *in vitro* transcription using a T7 RNA polymerase (Promega). Riboprobe was diluted in hybridization solution (50 % formamide, 10 mM Tris-HCl pH 8.0, 5 mg tRNA, 10 mM dithiothreitol/DTT, 10% dextran sulfate, 0.3 M NaCl, 1 mM EDTA, and 1× Denhardt’s solution), and brain slices were then hybridized overnight at 57°C. As post-hybridization, slides were incubated in 0.002% RNAse A for 30 minutes followed by stringent washes in SSC (sodium chloride-sodium citrate buffer). Sections were then dipped in NTB autoradiographic emulsion (Kodak) and stored in light-protected slide boxes at 4°C for 5 days. Signal was developed with developer and fixer (Carestream, Rochester, NY, USA), and cover-slipped with DPX (Electron Microscopy Sciences) mounting medium. Dark-field photomicrographs were acquired using an Axio Imager M2 (Carl Zeiss).

**Antibodies for Western blotting and immunostaining**

The following antibodies were used for Western blotting and immunostaining: Sel1L (rabbit, 1:2,000, ab78298; Abcam); BiP/GRP78 (goat, 1:1,000 for Western blotting and 1:200 for immunostaining; sc-1051; Santa Cruz Biotechnology Inc.); HSP90 (rabbit, 1:5,000, sc-13119, Santa Cruz Biotechnology Inc.); p62 (rabbit,1:1,000 for Western blotting and 1:500 for immunostaining; PM045, MBL); FAM134B (rabbit, 1:1,000,21537-1-AP, Proteintech); LC3 (rabbit, 1:1,000, #2775, Cell Signaling Technology); KDEL (rabbit, 1:500 for immunostaining, NBP1-97469, Novus Biologicals); CNX (rabbit,1:1,000, #2679 Cell Signaling Technology); XTP3B (goat, 1:500 for immunostaining; sc-161409, Santa Cruz Biotechnology Inc.); HA (mouse, 1:5000, H9658, Sigma-Aldrich); β-actin (mouse, 1:5000, A1978, Sigma-Aldrich); Flag (mouse, 1:5000, A8592, Sigma-Aldrich); c-Myc (mouse, 1:5000, #11814150001, Roche). Anti-Hrd1 antibody (rabbit, 1:300 for immunostaining and 1:500 for Western blotting) was provided by Richard Wojcikiewica (SUNY Upstate Medical University, Syracuse, New York, USA). Anti-NPII (mouse, 1:200 for immunostaining of proAVP and mature NPII, PS41) and anti-AVP (rabbit, 1:200 for immunostaining of proAVP and mature AVP, VA4) were provided by Harold Gainer (National Institute of Neurological Disorders and Stroke, NIH, Bethesda, Maryland, USA). Anti-H2A (rabbit, 1:10.000 for western blotting) was a gift of Yihong Ye (National Institute of Diabetes and Digestive and Kidney Disease, NIH). The secondary antibodies used for Western blotting were goat ant-rabbit IgG HRP and goat anti-mouse IgG HRP (1:5,000; both from Bio-Rad). Donkey anti-goat IgG was obtained from Jackson ImmunoResearch Laboratories. The secondary antibodies used for fluorescence immunostaining (all at 1:500) were anti-mouse IgG Alexa fluor 488, anti-rabbit Alexa Fluor 594 and Alexa Fluor 680 (Jackson ImmunoReserch). Detection of protein phosphorylation by phos-Tag was described previously [9].

**Immunoreactivity signal quantitation and colocalization analysis**

Fluorescent samples were imaged with a Nikon A1 confocal microscope, and imaging parameters were identical within each set of samples. Brain in-situ hybridization slides were scanned using an Aperio Scanscope (Leica Biosystems). To quantify cell body immunnostained or hybridized probe signal using ImageJ, individual cells were outlined, and fluorophore intensity was measured as average gray value (intensity/area). To quantify axon immunnostained intensity, equal areas were outlined, and fluorophore intensity was measured as above.

**Cell lines and transfection for analyzing proAVP maturation and plasmids**

HEK293T cells, N2a cells (both from ATCC) and Hela cells were cultured in DMEM (Corning, NY) with 1% Pen/Strip and non-treated or heat inactivated 10% FBS (GIBCO), respectively. Cells were transfected within 24 hours after plating with PEI (HEK293T cells) or Lipofectamine 2000 (N2a cells and Hela cells). To active starvation-induced autophagy, cells washed with PBS at least three times were incubated in EBSS (Gibco, 14155063) for the indicated time. Human WT, G57S proAVP plasmids and HRD1-Myc construct were described previously [7, 9]. The pcDNA3.1-Flag-TEX264,CALCOCO1, CCPG1,ATL3,CDK5RAP3,and FAM134B, were cloned from human cDNA samples and constructed by standard molecular biology techniques or described previously [10].

**CRISPR/Cas9 and siRNA for targeted genes**

Generation of HRD1-deficient HEK293T and N2a cells was previously described [7], and the generation of ATG5-KO HEK293T cells was described previously [11]. To generate Atg7-deficient N2a cells, the siRNA sequence for mouse Atg7 (5'GGGUUAUUACUACAAUGGUG-3′) was used [12]. For knocking-down of FAM134B in Hela cells, Chemically synthesized 21-nucleotide siRNA duplexes were synthesized (Sangon Biotech) and transfected using Lipofectamine RNAiMAX (Invitrogen, 13778030) according to the manufacturer’s instructions, with the following sequence: siRNA #1, 5′-AGCUAUCAAAGACCAGUUATT-3′; #2, forward: 5′-GCUCAGCCACUGUAUUGCAGA-3′; #3, 5′-CCUCUGAACAGUGACCAAATT-3′. #3 sequence showed the best knocking-down efficiency and was used for the following assays.

**Prediction of aggregation propensity in protein structures**

The aggregation propensity of proAVP was predicted via Aggrescan 3D 2.0 server (https://biocomp.chem.uw.edu.pl/A3D2/) through a job ID (https://biocomp.chem.uw.edu.pl/A3D2/hproteome_job/9b3fcd26911dc95/) as shown (**Fig. S2A-B**) [13].

**Statistics**

Results are expressed as the mean ±SEM unless otherwise stated. Comparisons between groups were made by *Student’s t-test* between two groups, by one-way ANOVA for multiple groups with single variable, and by two-way ANOVA for multiple groups with two variables*,* except for specifically indicated. p value of less than 0.05 was considered statistically significant, unless specifically indicated. All experiments were repeated at least 3 times or performed with independent experiments or biological samples, unless stated specifically, and representative data are shown.

**References**

1. Romanov, R.A., et al., *Molecular design of hypothalamus development.* Nature, 2020. 582(7811): p. 246-252.

2. Hao, Y., et al., *Integrated analysis of multimodal single-cell data.* Cell, 2021. 184(13): p. 3573-3587 e29.

3. Pauza, A.G., et al., *Osmoregulation of the transcriptome of the hypothalamic supraoptic nucleus: A resource for the community.* J Neuroendocrinol, 2021. 33(8): p. e13007.

4. Wu, T., et al., *clusterProfiler 4.0: A universal enrichment tool for interpreting omics data.* Innovation (Camb), 2021. 2(3): p. 100141.

5. Cho, K.F., et al., *Proximity labeling in mammalian cells with TurboID and split-TurboID.* Nat Protoc, 2020. 15(12): p. 3971-3999.

6. Feng, R., et al., *The rapid proximity labeling system PhastID identifies ATP6AP1 as an unconventional GEF for Rheb.* Cell Res, 2024. 34(5): p. 355-369.

7. Shi, G., et al., *ER-associated degradation is required for vasopressin prohormone processing and systemic water homeostasis.* J Clin Invest, 2017. 127(10): p. 3897-3912.

8. Shrestha, N., et al., *Integration of ER protein quality control mechanisms defines beta cell function and ER architecture.* J Clin Invest, 2023. 133(1).

9. Sun, S., et al., *IRE1a is an endogenous substrate of endoplasmic-reticulum-associated degradation.* Nat Cell Biol, 2015. 17(12): p. 1546-1555.

10. Hao, J., et al., *NLRC5 restricts dengue virus infection by promoting the autophagic degradation of viral NS3 through E3 ligase CUL2 (cullin 2).* Autophagy, 2023. 19(4): p. 1332-1347.

11. Chen, M., et al., *TRIM14 Inhibits cGAS Degradation Mediated by Selective Autophagy Receptor p62 to Promote Innate Immune Responses.* Mol Cell, 2016. 64(1): p. 105-119.

12. Wei, Y., et al., *EGFR-mediated Beclin 1 phosphorylation in autophagy suppression, tumor progression, and tumor chemoresistance.* Cell, 2013. 154(6): p. 1269-84.

13. Kuriata, A., et al., *Aggrescan3D (A3D) 2.0: prediction and engineering of protein solubility.* Nucleic Acids Res, 2019. 47(W1): p. W300-W307.
